# Understanding G × E Interaction for Nutritional and Antinutritional Factors in a Diverse Panel of *Vigna stipulacea* (Lam.) Kuntz Germplasm Tested Over the Locations

**DOI:** 10.3389/fpls.2021.766645

**Published:** 2021-12-13

**Authors:** Padmavati G. Gore, Arpita Das, Rakesh Bhardwaj, Kuldeep Tripathi, Aditya Pratap, Harsh K. Dikshit, Sudip Bhattacharya, Ramakrishnan M. Nair, Veena Gupta

**Affiliations:** ^1^Division of Plant Genetic Resources, Indian Council of Agricultural Research – Indian Agricultural Research Institute, New Delhi, India; ^2^Indian Council of Agricultural Research – National Bureau of Plant Genetic Resources, New Delhi, India; ^3^Bidhan Chandra Krishi Viswavidyalaya, Mohanpur, India; ^4^Indian Council of Agricultural Research – Indian Institute of Pulses Research, Kanpur, India; ^5^Division of Genetics, Indian Council of Agricultural Research – Indian Agricultural Research Institute, New Delhi, India; ^6^World Vegetable Center, South and Central Asia, Hyderabad, India

**Keywords:** GGE biplot, minerals, phytic acid, protein, underutilized legume, *Vigna stipulacea*

## Abstract

Micronutrient malnutrition or hidden hunger is a serious challenge toward societal well-being. *Vigna stipulacea* (Lam.) Kuntz (known locally as *Minni payaru*), is an underutilized legume that has the potential to be a global food legume due to its rich nutrient profile. In the present study, 99 accessions of *V. stipulacea* were tested for iron (Fe), zinc (Zn), calcium (Ca), protein, and phytate concentrations over two locations for appraisal of stable nutrient-rich sources. Analysis of variance revealed significant effects of genotype for all the traits over both locations. Fe concentration ranged from 29.35–130.96 mg kg^–1^ whereas Zn concentration ranged from 19.44 to 74.20 mg kg^–1^ across both locations. The highest grain Ca concentration was 251.50 mg kg^–1^ whereas the highest grain protein concentration was recorded as 25.73%. In the case of grain phytate concentration, a genotype with the lowest value is desirable. IC622867 (G-99) was the lowest phytate containing accession at both locations. All the studied traits revealed highly significant genotypic variances and highly significant genotype × location interaction though less in magnitude than the genotypic variance. GGE Biplot analysis detected that, for grain Fe, Zn, and Ca concentration the ‘ideal’ genotypes were IC331457 (G-75), IC331610 (G-76), and IC553564 (G-60), respectively, whereas for grain protein concentration IC553521 (G-27) was the most “ideal type.” For phytate concentration, IC351407 (G-95) and IC550523 (G-99) were considered as ‘ideal’ and ‘desirable,’ respectively. Based on the desirability index, Location 1 (Kanpur) was identified as ideal for Fe, Zn, Ca, and phytate, and for grain protein concentration, Location 2 (New Delhi) was the ideal type. A significant positive correlation was detected between grain Fe as well as grain Zn and protein concentration considering the pooled analysis over both the locations where as a significant negative association was observed between phytate and protein concentration over the locations. This study has identified useful donors and enhanced our knowledge toward the development of biofortified *Vigna* cultivars. Promoting domestication of this nutrient-rich semi-domesticated, underutilized species will boost sustainable agriculture and will contribute toward alleviating hidden hunger.

## Introduction

Micronutrient deficiency (MNDs) or “Hidden hunger” is considered a global crisis affecting more than 2 billion people in the developing countries of South Asia, Africa, and Latin America (Bouis et al., 2013; Kumssa et al., 2015; Migliozzi et al., 2015; Wakeel et al., 2018). It was estimated of the major micronutrients that 60% of the world’s population is iron (Fe) deficient, over 30% are zinc (Zn) deficient, and 12.2% are protein deficient (Food and Agriculture Organization of the United Nation [FAO], 2014; Stein, 2014). The deficiencies of other essential micronutrient components of the diet, including calcium (Ca), are also prevalent and ultimately hamper the well-being of humanity (Kumssa et al., 2015; Beal et al., 2017; Narayanam et al., 2021). Therefore, pressing needs toward reducing MNDs through biofortification was considered a key component by the United Nations in their Millennium Development Goals (MDGs) program. Systematic breeding efforts for improving the nutrient status and the development of biofortified varieties offer great scope for mitigating MNDs judiciously. As a prerequisite, substantial variability should be available in the germplasm set in terms of grain micronutrient concentrations as well as knowledge regarding inheritance pattern and genetic relationship of these micronutrients in the targeted crop (Upadhyaya et al., 2012, 2016; Velu et al., 2014; Phuke et al., 2017).

Pulses are a major source of plant-based protein and other nutrients like phosphorus, vitamins, minerals, riboflavin, and essential amino acids. The genus *Vigna savi* is one of the most important genera among all the pulse crops, containing more than 200 domesticated and wild species (Pratap et al., 2015). Wild *Vigna* species possess a high potential for utilization as human food and fodder for animals (Popoola et al., 2015). Some of them are already utilized as human food, for example, *V. marina* (Burm.) Merr. is used in Australia (Tomooka et al., 2011), *V. vexillata* (L.) A. Rich. in Southern Asia (Karuniawan et al., 2006; Tripathi et al., 2021), *V. racemosa* (G. Don) Hutch & Dalzeil. in Nigeria (Folashade et al., 2017), and *V. stipulacea* (Lam.) Kuntz. and *V. trilobata* (L.) Verdc. in Asia (Siddhuraju et al., 1992; Bravo et al., 1999; Gore et al., 2019). However, the utilization of wild *Vigna* species as food and fodder is still very restricted because of unawareness around their potential. Increased scientific efforts to explore the hidden potential of wild *Vigna* species are recommended while planning future food strategies (Harouna et al., 2018). The nutritional composition plays a crucial role in food acceptability and food preferences as it is directly linked to consumers’ health and well-being. *V. stipulacea*, known locally as *Minni payaru*, is being utilized in the southern part of India, mainly in Tamil Nadu, for animal feeding, manure production, and in some traditional dishes like “*Idli*” and “*Vada*” (Tomooka et al., 2011; Gore et al., 2021). Unfortunately, *V. stipulacea* is an under-researched legume and no reports are available to date regarding the variability of minerals *viz*., Fe, Zn, Ca, etc., and the protein concentration in the grains of this species. Mineral and micronutrient concentrations in many major crops reported quantitative inheritance with low heritability, thus confirming the convoluted role of soil composition and other environmental factors toward mineral availability (Stangoulis et al., 2007; Shi et al., 2008). Therefore, a holistic approach for deciphering genotype × environment interaction (henceforth GEI) is necessary to identify stable micronutrient-rich genotypes. Additionally, breeders are also willing to detect target test locations for conducting future screening programs. This species of *Vigna* is generally cultivated in dry areas with a rainfed ecosystem where substantial variation is observed regarding soil micronutrient status. Therefore, GEI for grain nutrient concentration is expected to be significant and treated as a step toward developing stable genotypic performance across environments.

Many statistical tools are available to measure the confounding role of environment followed by characterizing and grouping genotypes and environments. Among them, the GGE biplot is gaining popularity over other biplot analyses. This methodology was proposed by Yan et al. (2000), which highlighted that both genotype (G) and genotype × environment (GE) are the two sources of variation that should be considered concurrently for evaluation of genotypes and test environments precisely (Yan and Tinker, 2005). The beauty of this method in comparison to other biplot analyses is that it excludes the major influence of the environment (E) and considers the main genotypic effect coupled with the GEI effect in a lucid way for the evaluation of genotypes and testing locations (Yan et al., 2007; Yan and Holland, 2010). In earlier studies for comprehending GEI in food legumes concerning micronutrient availability, several statistical tools were used such as Eberhart and Russell (1966)’s method in peanut (Upadhyaya et al., 2012); mungbean (Singh et al., 2013); lentil (Kumar et al., 2013, 2014; Darai et al., 2020); or by using AMMI biplot in cowpea (da Silva and Santos, 2017) and lentil (Singh et al., 2017). Recently, Gupta et al. (2020) have deployed GGE biplot analysis for deciphering GEI toward identifying stable genotype in urdbean. To the best of our knowledge, this is the first report of expanding this methodology for precise appraisal of *V. stipulacea* genotypes concerning grain micronutrients and antinutritional factors. Therefore, keeping these in the backdrop, the present study was deliberated toward assessment of genetic variability of a diverse set of *V. stipulacea* genotypes concerning nutrients (Fe, Zn, Ca, and protein) and antinutritional factors (phytate) followed by determination of the GEI interaction for appraisal of nutrient-rich stable genotypes through GGE biplot approach. The findings of this study will make it easier for global researchers to select nutrient-rich *V. stipulacea* germplasm for future breeding and genomic studies.

## Materials and Methods

A total of 99 accessions of *V. stipulacea* were grown in the *Kharif* (monsoon season) of 2018–19 at two locations. The details of the accessions and their geographical origin are listed in Table 1. Hereafter, the two testing locations are referred to as Loc1 for ICAR-Indian Institute of Pulses Research (IIPR), Kanpur located at 26°27′N latitude, 80°14′E longitude, 152.4 m above mean sea level (AMSL), and Loc2 for ICAR-National Bureau of Plant Genetic Resources (NBPGR), New Delhi, located at a latitude of 28°40′N and longitude of 77°12′E and an altitude of 228 m AMSL.

**TABLE 1:** Details of accessions and their geographical origin.

S. no.	Accession no.	Genotype no.	Village	District	State
1.	IC252016	G1	Nandikotkur	Kurnool	Andhra Pradesh
2.	IC261321	G2	Nandikotkur	Kurnool	Andhra Pradesh
3.	IC261384	G3	Ramachandrapuram	Kurnool	Andhra Pradesh
4.	IC305192	G4	Unknown	Kurnool	Andhra Pradesh
5.	IC553494	G5	Atmakur	Kurnool	Andhra Pradesh
6.	IC610275	G6	Nallavagulapalle	Kurnool	Andhra Pradesh
7.	IC524667	G7	Mydukuru	Cuddapah	Andhra Pradesh
8.	IC550531	G8	Kothavalasa	Vizianagaram	Andhra Pradesh
9.	IC550532	G9	S.Kota	Vizianagaram	Andhra Pradesh
10.	IC550533	G10	Narsipatnam	Vizianagaram	Andhra Pradesh
11.	IC550536	G11	Vajragadda	Vizianagaram	Andhra Pradesh
12.	IC550538	G12	Anakapalle	Vizianagaram	Andhra Pradesh
13.	IC550545	G13	Amadalavalasa	Srikakulam	Andhra Pradesh
14.	IC550548	G14	Regolu	Srikakulam	Andhra Pradesh
15.	IC550551	G15	Panukuvalasa	Srikakulam	Andhra Pradesh
16.	IC550553	G16	Lainakothuru	Vishakhapatnam	Andhra Pradesh
17.	IC553502	G17	Kurchintalabhai	Mahbubnagar	Andhra Pradesh
18.	IC524639	G18	Karedu	Prakasam	Andhra Pradesh
19.	IC553505	G19	Buddareddypalle	Prakasam	Andhra Pradesh
20.	IC553509	G20	Buddareddypalle	Prakasam	Andhra Pradesh
21.	IC553510	G21	Buddareddypalle	Prakasam	Andhra Pradesh
22.	IC553512	G22	Vepagumpalle	Prakasam	Andhra Pradesh
23.	IC553516	G23	Neredupally	Prakasam	Andhra Pradesh
24.	IC553517	G24	Neredupally	Prakasam	Andhra Pradesh
25.	IC553518	G25	Neredupally	Prakasam	Andhra Pradesh
26.	IC553520	G26	Bonduru	Prakasam	Andhra Pradesh
27.	IC553521	G27	Bonduru	Prakasam	Andhra Pradesh
28.	IC553522	G28	Konijeyedu	Prakasam	Andhra Pradesh
29.	IC553523	G29	Konijeyedu	Prakasam	Andhra Pradesh
30.	IC553524	G30	M.Nedemalluru	Prakasam	Andhra Pradesh
31.	IC553525	G31	M.Nedemalluru	Prakasam	Andhra Pradesh
32.	IC553526	G32	M.Nedemalluru	Prakasam	Andhra Pradesh
33.	IC553534	G33	Bollapalle	Prakasam	Andhra Pradesh
34.	IC553535	G34	Jontali	Prakasam	Andhra Pradesh
35.	IC553527	G35	Musnoor	Nellore	Andhra Pradesh
36.	IC553528	G36	Budamagunta	Nellore	Andhra Pradesh
37.	IC553529	G37	Nellore	Nellore	Andhra Pradesh
38.	IC553530	G38	Nellore	Nellore	Andhra Pradesh
39.	IC553531	G39	Nellore	Nellore	Andhra Pradesh
40.	IC553532	G40	Nellore	Nellore	Andhra Pradesh
41.	IC553537	G41	Kornipadu	Krishna	Andhra Pradesh
42.	IC553538	G42	Kornipadu	Krishna	Andhra Pradesh
43.	IC553539	G43	Pamulapadu	Krishna	Andhra Pradesh
44.	IC553540	G44	Pamulapadu	Krishna	Andhra Pradesh
45.	IC553541	G45	Ramanapudi	Krishna	Andhra Pradesh
46.	IC553544	G46	Gudivada	Krishna	Andhra Pradesh
47.	IC553547	G47	Peddavogirala	Krishna	Andhra Pradesh
48.	IC553548	G48	Peddavogirala	Krishna	Andhra Pradesh
49.	IC553551	G49	Tadanki	Krishna	Andhra Pradesh
50.	IC550520	G50	Tanuku	West Godavari	Andhra Pradesh
51.	IC553553	G51	Chudimella	West Godavari	Andhra Pradesh
52.	IC553554	G52	Kuppalakunta	West Godavari	Andhra Pradesh
53.	IC553555	G53	Nallajerla	West Godavari	Andhra Pradesh
54.	IC553556	G54	Achannapalem	West Godavari	Andhra Pradesh
55.	IC553557	G55	Chodavaram	West Godavari	Andhra Pradesh
56.	IC553558	G56	Chebrol	West Godavari	Andhra Pradesh
57.	IC553560	G57	Badampudi	West Godavari	Andhra Pradesh
58.	IC553561	G58	Peddatapdenalle	West Godavari	Andhra Pradesh
59.	IC553562	G59	Peddatadepalle	West Godavari	Andhra Pradesh
60.	IC553564	G60	Chinnatadepallegudem	West Godavari	Andhra Pradesh
61.	IC553565	G61	Bangarugudem	West Godavari	Andhra Pradesh
62.	IC622860	G62	KVK Campus Rewa	Rewa	Madhya Pradesh
63.	IC622861	G63	Khairan	Rewa	Madhya Pradesh
64.	IC276983	G64	Raisen	Raisen	Madhya Pradesh
65.	IC622865	G65	Naibag(ICAR)	Bhopal	Madhya Pradesh
66.	IC210580	G66		Thrissur	Kerala
67.	IC251435	G67	Junagadh	Junagadh	Gujarat
68.	IC024837	G68		East Godavari	Andhra Pradesh
69.	IC251436	G69			Odisha
70.	IC331436	G70	Jiban Deipur	Khurda	Odisha
71.	IC331437	G71	Lanja	Ganjam	Odisha
72.	IC331453	G72	IGAU Campus Raipur	Raipur	Chattisgarh
73.	IC331454	G73	Ghumia	Raipur	Chattisgarh
74.	IC331456	G74	Sariah	Bilaspur	Chattisgarh
75.	IC331457	G75	Sariah	Bilaspur	Chattisgarh
76.	IC331610	G76	Sariah	Bilaspur	Chattisgarh
77.	IC251438	G77		Coimbatore	Tamil Nadu
78.	IC349701	G78	Anamali	Coimbatore	Tamil Nadu
79.	IC351406	G79	Valathi	Villupuram	Tamil Nadu
80.	IC417392	G80	Olangkenaru	Coimbatore	Tamil Nadu
81.	IC622867	G81	Somarasanpettai/Adavathur	Trichy	Tamil Nadu
82.	IC622868	G82	Kavakkaranpatti	Trichy	Tamil Nadu
83.	IC622869	G83	Pothavur	Trichy	Tamil Nadu
84.	IC521211	G84	varichur	Madurai	Tamil Nadu
85.	IC521245	G85	Mullaikaraipatti	Trichy	Tamil Nadu
86.	IC521215	G86	Chatrakudi	Ramanathapuram	Tamil Nadu
87.	IC259512	G87	Farmagudi	North Goa	Goa
88.	IC037804	G88			Tamil Nadu
89.	IC622870	G89	Pothavur	Trichy	Tamil Nadu
90.	IC550524	G90	Tadepallegudem	West Godavari	Andhra Pradesh
91.	IC421767	G91	Byahatti	Belgaum	Karnataka
92.	IC024830	G92			Karnataka
93.	IC331450	G93		Malkangiri	Odisha
94.	IC625694	G94	Cheruthani	Idukki	Kerala
95.	IC351407	G95			Tamil Nadu
96.	IC406517	G96			Tamil Nadu
97.	IC467707	G97	Dhoregaon/Gangapur	Aurangabad	Maharashtra
98.	IC550522	G98	Tadepallegudem	West Godavari	Andhra Pradesh
99.	IC550523	G99	Tadepallegudem	West Godavari	Andhra Pradesh

At both locations, accessions were sown under natural field conditions. The recommended package of practices for growing *Kharif Vigna* species was followed. For meeting the balanced nutrient demand of the crop, 100 kg ha^–1^ Di-ammonium Phosphate (DAP) was applied as a basal dose before sowing. One pre-sowing irrigation was given to ensure proper germination in the field, while light irrigation was also given at the flowering stage. Sufficient moisture was available during the entire cropping season at both locations. The mechanical scarification of seeds was done to ensure maximum germination (Pratap et al., 2015; Singh N. et al., 2020). At ICAR-IIPR, accessions were grown in customized pots in a *Vigna* wide hybridization garden. Each pot measured 1 m in diameter and was about 1 m high. Further, each pot had provision of drainage at the bottom and sides so that water stagnation did not occur during excess precipitation. In each accession, 20 seeds were sown in individual pots around the periphery maintaining an equal distance of 5–7 cm between two plants. At ICAR-NBPGR, every accession was grown in two rows, each with 4-m length, with a row-to-row spacing of 60 cm. No insecticide was sprayed and manual weeding was done at both locations 25–30 days after sowing.

### Soil Environment of the Test Locations

Geographically, experimental location 1 (Loc1, ICAR-IIPR, Kanpur) falls under the subtropical zone in the Indo-Gangetic Plains. The experimental site was well-drained, and the soil type is silty clay loam, slightly alkaline inceptisol. The climate is tropical sub-humid with an annual rainfall of 722 mm and mean annual maximum and minimum temperatures of 33°C and 20°C, respectively. The experimental site of location 2 (Loc2, ICAR-NBPGR, New Delhi) was well-drained, and the soil was sandy loam and slightly alkaline (pH 7.8). The climate is tropical sub-humid with an annual rainfall of 750 mm and mean annual maximum and minimum temperatures of 31°C and 17.3°C, respectively.

### Estimation of Minerals (Fe, Zn, and Ca) in Seeds of *Vigna stipulacea*

The seeds were collected at the harvesting stage and sorted by removing damaged seeds and foreign materials. The pure and clean seeds were analyzed for iron (Fe), zinc (Zn), and calcium (Ca). All the chemicals, including standards used in the present study, were of high purity. Mineral concentrations were determined by following the official analytical methods (AOAC, 2005). Powdered grain samples weighing 0.5 g were transferred to a silica basin for ashing. Silica basins were kept in the muffle furnace at 300°C with the temperature gradually increased to 500–600°C for 5–6 h until the powder turned into ash. Next, 10 ml of dilute HCI and 50 ml of water were added to the ash and kept on the bath until all salt was diluted and a crystal-clear solution was obtained. The solution was filtered through Whatman No. 44 (ashless) filter paper, and the filtrate was collected in the volumetric flask. Elemental analysis of Fe, Zn, and Ca were carried out using collected filtrate with atomic absorption spectrometer (AAS, model-Varian Spectra AA 220 FS, Varian Australia Pty Ltd., Australia) equipped with a D2 lamp background correction system using an air-acetylene flame. Five blank samples containing nitric acid and perchloric acid (4:1) were also digested simultaneously with the samples. Calibration of the instrument was done with specific standards for each element.

### Estimation of Total Protein in Seeds of *Vigna stipulacea*

Total protein was estimated as per the AOAC official method with some modifications (AOAC, 2005). Dried seeds of all the accessions were grounded and 0.1 g powdered material was digested with digestion mixture (made of sulfuric acid, anhydrous sodium sulfate, selenium, and hydrogen peroxide) in glass digestion tubes at 420°C, until it was converted into a crystal clear solution. The nitrogen percentage in the solution was estimated by Kjeltech (FOSS Tecator) nitrogen auto-analyzer. To ascertain recovery, in-house QC samples and food reference material ASFRM 14 were used as control. A recovery percentage of 99.8 ± 1.6 for AS-FRM 14 and 102.7 ± 1.2 for QC samples were obtained.

### Estimation of Phytic Acid in Seeds of *Vigna stipulacea*

The pure and clean seeds were used to measure phytate and free phosphorous concentration with the help of phytic acid/total phosphorous assay kit (K-PHYT) from Megazyme, Ireland. Recovery of (95.2% ± 1.4) for control oat flour was obtained, and results were expressed as g 100 g^–1^ sample. Supplementary Figure S1 represented the flow chart of material and method.

### Calculation of % Recommended Daily Allowance

The % recommended daily allowance (RDA) was calculated as per DeFries et al. (2015). It was done by taking into account the % requirement of Fe, Zn, Ca, and protein by consuming 100 g of *V*. *stipulacea* seeds to meet the nutritional requirements of a healthy person of a given sex, age, life stage, or physiological condition (adolescence stage, pregnancy, etc.) in male and female consumers. According to the Institute of Medicine (US) Panel on Micronutrients (2001), RDA per day per person for Fe is 8 mg and 18 mg for male and female, respectively whereas for Zn it is 11 and 8 mg for male and female, respectively. In the case of Ca, the RDA per day per person is 1000 mg for both sexes where as, for protein, the % RDA is 56 g for an adult man and 46 g for an adult woman. This % RDA was calculated for the female/male belonging to the 9 to 50 years age group.

### Statistical Analysis

Combined analysis of variance (ANOVA) for each parameter (Fe, Zn, Ca, protein, and phytate) was performed to elucidate the significant effects of G, E, and GEI across the locations using R Studio application (R Development Core Team, 2012). Genetic parameters *viz*., phenotypic coefficient of variation (PCV) and genotypic coefficient of variation (GCV) as well as heritability for all the studied traits were estimated using the standard procedure (Singh and Chaudhary, 1979). The genetic advance was estimated following the method proposed by Allard (1960). Finally, the stability of the tested genotypes over the locations was enumerated and portrayed graphically by deploying GGE biplot analysis (Yan, 2001). In GGE biplot, the first principal component (PC1) scores of the genotypes and the environments concerning the values of four micronutrients (Fe, Zn, Ca, and protein) and the phytate concentration was plotted against the respective scores for the second principal component (PC2) originating from Singular Value Decomposition (SVD) of environment-centered data that was explained in detail by Yan and Kang (2003) with the following equation:


(1) $$Y_{\text{ij}}=\mu +e_{\text{j}}+\sum_{n=1}^{N}\lambda_{\text{n}}\gamma_{\text{in}}\delta_{\text{jn}}+\varepsilon_{\text{ij}}$$

where,

*Y*_ij_ = mean response of *i^th^* genotype (*i* = 1, …, *I*) in the *j^th^* environment (*j* = 1, …, *J*)

μ = grand mean

*e*_j_ = environment deviations from the grand mean

λ_*n*_ = the eigen value of PC analysis axis

γ_in_ and δ_jn_ = genotype and environment principal components scores for axis *n*

*N* = number of principal components retained in the model and

ε_ij_ = residual effect∼ *N* (0,σ^2^)

The data from both locations were computed without scaling to generate a tester-centered (centering 2) GGE biplot as suggested by Yan and Tinker (2006). Regarding appraisal of the testing genotypes, genotype-focused singular value partitioning (SVP = 1) was used for generating “mean vs. stability” graph, whereas, in the case of evaluation of testing locations, environment-focused singular value partitioning (SVP = 2) was deployed (Yan, 2001) using “discriminating power vs. representativeness” biplot view. Moreover, the “desirability index” of the testing location was calculated to assess the superiority of the testing locations, which is a combined index of “discriminating power” and ‘representativeness’ following the standard method (Yan, 2014).

## Results

### Descriptive Statistics of *Vigna stipulacea* Genotypes Regarding Grain Micronutrients and Phytate Concentration

**FIGURE 1:**
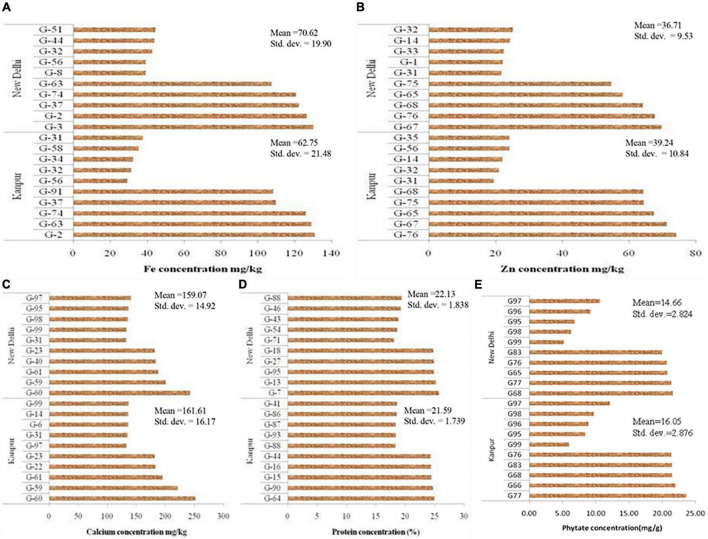
Mean value of five highest and lowest accession of *Vigna stipulacea* regarding nutrients and antinutrient concentration in grain. **(A)** Fe concentrations at Loc1 and Loc2. **(B)** Zn concentrations at Loc1 and Loc2. **(C)** Ca concentrations at Loc1 and Loc2. **(D)** Protein concentrations at Loc1 and Loc2. **(E)** Phytate concentrations at Loc1 and Loc2.

Mean performance regarding grain micronutrients (Fe, Zn, Ca, and protein) and phytate concentration of 99 *V. stipulacea* genotypes are presented in Supplementary Table S1. Grain micronutrients concentration is highly variable and influenced by genotypic differences for their acquisition, mobilization, and further accumulation in grain. Besides genotypic differences, environmental factors like soil properties and fertility status are also the determining factors to influence micronutrient concentrations in the grain. The mean performance of the five best performing and five least performing genotypes regarding grain Fe concentration for Loc1 ranged from 29.4 mg kg^–1^ (G-56) to 131 mg kg^–1^ (G-2), whereas in the case of Loc2, it varied between 39.1 mg kg^–1^ (G-8) to 130 mg kg^–1^ (G-3) (Figure 1). Regarding grain Zn concentration, the highest Zn containing genotype in Loc1 was G-76 (74.2 mg kg^–1^), whereas, in Loc2, it was G-67 (69.8 mg kg^–1^). The presence of cross-over interaction (COI) was observed for grain Fe and Zn concentration as genotypes positions changed over the locations. The grain Ca concentration was the highest in G-60 with 252 mg kg^–1^ to 243 mg kg^–1^ at Loc1 and Loc2, respectively. In the case of grain protein concentration, G-64 was the highest protein-containing genotype (25.0%) at Loc1, whereas at Loc2, G-7 with 25.7% protein was the best one. The presence of COI was also observed in the case of grain protein concentration. With reference to phytate concentration, a genotype with the lowest value is desirable. It was detected that G-99 was the lowest phytate containing genotype at both the locations, with values ranging from 5.20 mg g^–1^ to 6.05 mg g^–1^ at Loc2 and Loc1, respectively.

The average grain Fe concentration of the tested *V. stipulacea* genotypes over both locations was 66.7 mg kg^–1^, whereas the average Zn concentration was 38.0 mg kg^–1^ (Table 2). The average Ca concentration over both locations was 160 mg kg^–1^. In the case of protein concentration, an average value of 21.9% was observed over both locations. An average phytate concentration of 15.4 mg g^–1^ was detected among the tested *V. stipulacea* genotypes over both locations. The highest PCV and GCV were detected in grain Fe concentration (29.4 and 29.2), whereas the lowest PCV and GCV were observed in protein concentration (6.28 and 5.95). The estimates of GCV and PCV were high (>20%) for Fe and Zn concentration only.

**TABLE 2:** Descriptive statistics regarding grain nutrients [Fe, Zn, Ca, and protein, and antinutrient (phytate)] concentration of *Vigna stipulacea*.

Characters	Grand mean	GCV	PCV	Heritability (%)	Genetic advance	Genetic advance as % of mean
Fe (mg kg^–1^)	66.7	29.2	29.4	98.5	39.8	59.7
Zn (mg kg^–1^)	38.0	24.8	25.0	98.5	19.3	50.8
Ca (mg kg^–1^)	160	9.40	9.72	93.4	30.0	18.7
Protein (%)	21.9	5.95	6.28	89.9	2.54	11.6
Phytate (mg g^–1^)	15.4	17.8	18.1	97.5	5.60	36.3

Heritability ranged from 89.9% in protein concentration to 98.5% in grain Fe and Zn concentration. High heritability tied with High GA was observed for almost all characters except protein concentration. The pooled ANOVA exhibited that the effect of genotypes, environment, and the GEI effects were significant for all the five traits at both locations (Table 3).

**TABLE 3:** Combined Analysis of variance for grain nutrients and antinutritional factors over two locations in *V. stipulacea* accessions.

Source of variation	DF	Mean sum of square				
		Fe	Zn	Protein	Ca	Phytate
ENV	1	9198.97	951.67	43.00	951.39	285.35
GEN	98	2423.43	579.79	14.68	1407.91	46.9
ENV × GEN	98	149.60	46.08	4.52	45.43	1.85

**FIGURE 2:**
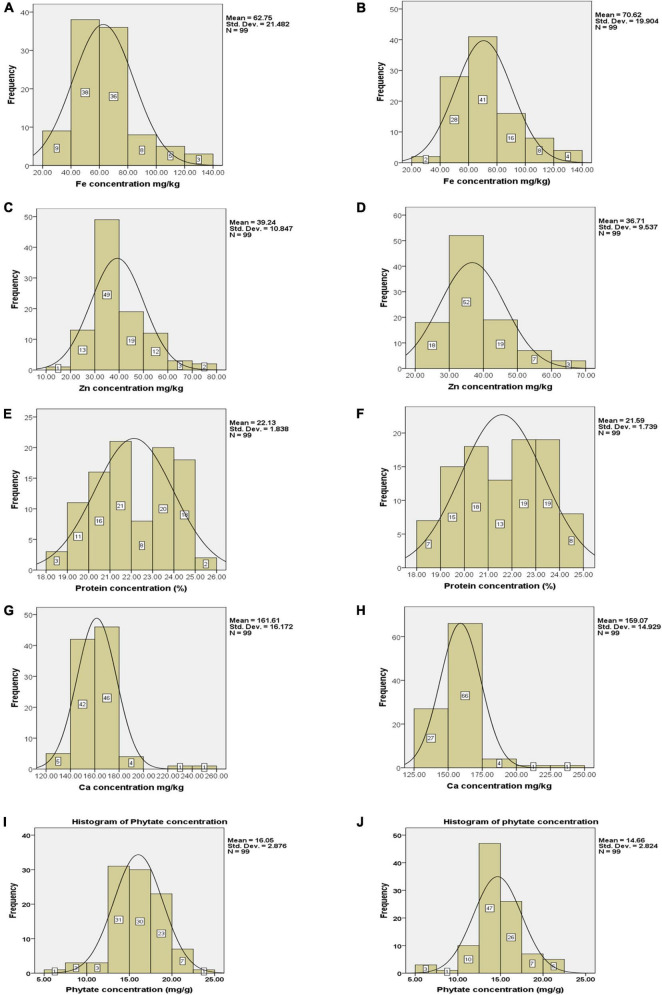
Frequency distribution of Fe, Zn, Ca, protein, and phytate concentrations **(A–J)** in tested *Vigna stipulacea* accessions at Loc1 (ICAR-IIPR, Kanpur); and Loc2 (ICAR-NBPGR, New Delhi), respectively. **(A)** Fe concentrations at Loc1. **(B)** Fe concentrations at Loc2. **(C)** Zn concentrations at Loc1. **(D)** Zn concentrations at Loc2. **(E)** Ca concentrations at Loc1. **(F)** Ca concentrations at Loc2. **(G)** Protein concentrations at Loc1. **(H)** Protein concentrations at Loc2. **(I)** Phytate concentrations at Loc1. **(J)** Phytate concentrations at Loc2.

Frequency distribution regarding grain Fe concentration revealed that only 16 genotypes had Fe concentration between 80 and 140 mg kg^–1^ in Loc1 whereas, in Loc2, 28 genotypes were grouped in this range (Figures 2A,B). There were only seven genotypes with Fe concentration in the range of 20–40 mg kg^–1^. In the case of Zn concentration, it was observed that a large number of genotypes were categorized in the range of 30–40 mg kg^–1^ of Zn in Loc1, and only five genotypes had Zn concentration within the range of 60–80 mg kg^–1^. In Loc2, 18 genotypes had Zn concentration in the range of 20–30 mg kg^–1^, 52 genotypes were showing Zn concentration between 30 and 40 mg kg^–1^, 19 genotypes in between 40 and 50 mg kg^–1^, seven genotypes in between 50 and 60 mg kg^–1^, and only three genotypes had more than 60 mg kg^–1^ of Zn concentration (Figures 2C,D). A well-distributed frequency graph was obtained regarding protein concentration of 99 *V. stipulacea* genotypes across both locations. In Loc1, only two genotypes with more than 25.0% protein concentration were detected, whereas, in Loc2, eight genotypes with protein concentration in the range of 24.0–25.0% were observed (Figures 2E,F). In the case of Ca concentration, it was detected that in Loc1, most genotypes were showing Ca concentration within the range of 140–180 mg kg^–1^, whereas in the case of Loc2, the highest frequency of genotypes was detected within the range of 150–175 mg kg^–1^ (Figures 2G,H). In the case of phytate concentration in Loc1, only one genotype exhibited a phytate value of 5.0 mg g^–1^, whereas, in the case of Loc2, three genotypes were detected within this range (Figures 2I,J).

**FIGURE 3:**
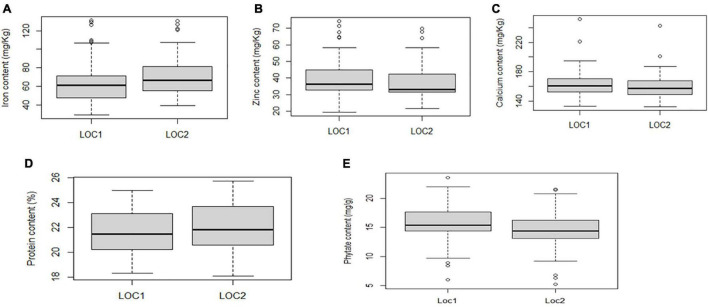
Boxplot view illustrating the distribution of Fe, Zn, Ca, protein, and phytate components across the two test locations. The upper and lower error bars represent the non-outlier range of the data set. The box represents the area from the first quartile to the third quartile. A horizontal line goes through the box at the median. The whiskers (vertical line) go from each quartile to the minimum or maximum. **(A)** Distribution of Fe concentration across two locations. **(B)** Distribution of Zn concentration across two locations. **(C)** Distribution of Ca concentration across two locations. **(D)** Distribution of protein concentration across two locations. **(E)** Distribution of phytate concentration across two locations.

Boxplot analysis depicted genotypes with the highest and lowest performance in five studied traits. The present study revealed that the median values for grain Fe and Zn concentration varied more between the locations than other traits. However, Ca concentration exhibited almost consistent median values between the locations (Figure 3).

### Percent Recommended Daily Allowance for Grain Micronutrients

Availability of RDA for grain Fe, Zn, Ca, and protein concentration from a serving of 100 g of *V. stipulacea* seeds as the food was calculated for each genotype across the locations (Supplementary Table S2). RDA for every nutrient differs with sex, so it was calculated separately for adult and consumers. It was observed that in Loc1, % RDA regarding Fe concentration for adult men ranged from 36.7 to 164% and 16.3 to 72.8% for an adult female. Similarly, in Loc2, the % RDA of Fe for adult male and female was 48.9–163% and 21.7–72.3%, respectively. In Loc1, the % RDA for Zn in the case of adult men ranged from 17.7 to 67.5%, whereas for female it was 24.3–92.8%, with an average value of 49.1%. However, in Loc2, the % RDA of Zn was 19.8–63.5% and 27.2–87.3% for an adult male and female, respectively. Regarding Ca concentration, the % RDA was meager compared to Fe and Zn and it was in the range of 1.33–2.52% and 1.33–2.43% for both male and female in Loc1 and Loc2, respectively. The % RDA of protein in Loc1 was 32.7–44.6% and 39.8–54.3% for adult male and female, respectively. In Loc2, it was 32.3- 46.0% and 39.4–55.9% for male and female, respectively.

### Effect of Environment on Grain Micronutrients and Phytate Concentrations

Grain micronutrient (Fe, Zn, Ca, and protein) concentration along with phytate concentration in all the tested genotypes revealed highly significant genotypic variances in all individual environments (data not shown) as well as in both the environments (Table 4). Grain phytate concentration exhibited the highest genotypic variance, followed by grain Fe concentrations. All the studied traits showed highly significant genotypic variances and highly significant genotype × location (σ^2^gl) interaction though less in magnitude than the genotypic variance.

**TABLE 4:** Genotypic variance (σ2g) and genotype × location interactions (σ2gl) for traits over the locations.

Characters	σ^2^g	σ^2^gl	Residual variance
Fe	378.97**	47.97**	5.69
Zn	88.95**	14.92**	1.33
Ca	227.08**	9.84**	15.92
Protein	1.69**	1.44**	0.19
Phytate	7508.99**	549.75**	192.70

**P < 0.01.

### Appraisal of Genotypes Based on Mean Performance and Stability Across the Locations

**FIGURE 4:**
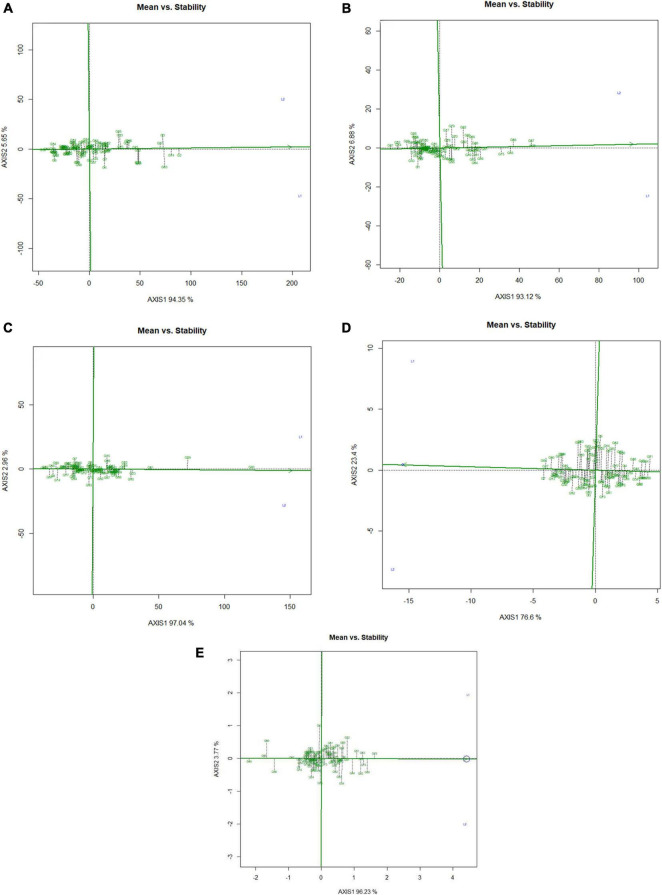
Mean vs. stability view of the GGE biplot over two locations. There was no transformation of data (transform = 0), and data were centered by means of the environments (centering = 2). The biplot was based on ‘row metric preserving.’ Numbers denote the serial numbers of genotypes as listed in Supplementary Table S1. Loc1 – ICAR-IIPR, Kanpur and; Loc2 – ICAR-NBPGR, New Delhi. **(A)** Mean vs. stability view of the GGE biplot regarding Fe concentration over two locations. **(B)** Mean vs. stability view of the GGE biplot regarding Zn concentration over two locations. **(C)** Mean vs. stability view of the GGE biplot regarding Ca concentration over two locations. **(D)** Mean vs. stability view of the GGE biplot regarding protein concentration over two locations. **(E)** Mean vs. stability view of the genotypes regarding phytate concentration over two locations.

**FIGURE 5:**
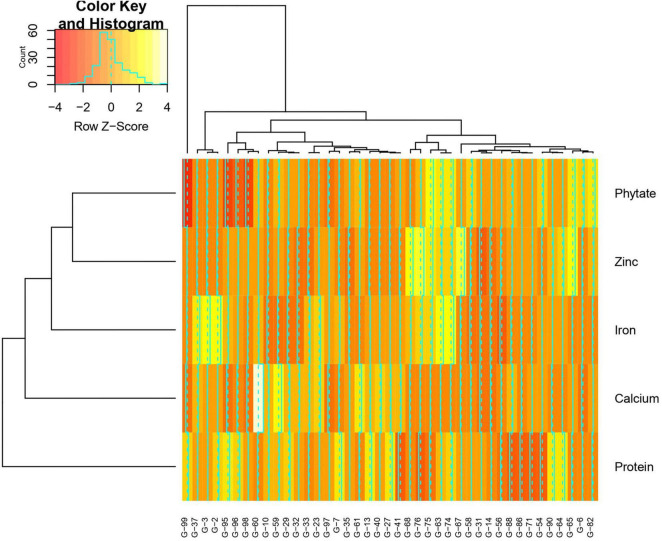
Hierarchical cluster analysis showing the relationship between the 5 highest and 5 lowest accessions of *Vigna stipulacea* for Fe, Zn, Ca, protein, and phytate over two locations (Loc1 – ICAR-IIPR, Kanpur, and Loc2 – ICAR-NBPGR, New Delhi). All together 41 accessions were considered for these five parameters. Numbers correspond to accessions as listed in Table 1.

Mean performance and genotypes stability were graphically portrayed through the “mean vs. stability” view of the GGE biplot, which represented both the mean performance and consistency of the genotypes in terms of grain micronutrients and phytate across the locations. This signifies that an ideal genotype should exhibit the least interaction with environmental factors. It can be judged by the “average environment coordination” (AEC) view of the GGE biplot (Yan, 2001) where environment centered (centering = 2) genotype-metric (SVP = 1) for grain Fe, Zn, Ca, protein, and phytate have been presented in Figures 4A–E, respectively. For grain Fe concentration, the first PC, i.e., PC1, explained 94.5%; for grain Zn concentration, the PC1 explained 93.1%; for grain Ca, the PC1 explained 97.0%; for protein concentration the PC1 explained 76.6% and for phytate concentration, PC1 explained 96.2% of the total variation. For all these traits, the cumulation of % contribution of both the PCs could explain total variations. In all these graphs, the single arrow headline passing through the biplot origin is known as “AEC abscissa,” representing the direction of higher mean values of grain micronutrients *viz*., Fe, Zn, Ca, and protein as well as phytate concentration of the genotypes. In addition, the double arrowed line perpendicular to the “AEC abscissa” represented “AEC ordinate.” With greater projection length of the “AEC ordinate” denoted less stability of the genotype’s performance and *vice versa*. Therefore, the average performance of the genotypes was resembled by the “AEC abscissa” projections of each genotype. Accordingly, it was detected that G-2, G-74, G-3, G-63, G-87, and G-82 were the best performing genotypes in terms of grain Fe concentration as these genotypes were placed toward the direction of “AEC abscissa.” On the other hand, G-56 was the lowest Fe containing genotype. It was observed that G-2, G-74, G-3, G-63, and G-87 were not stable genotypes, having greater projection value from the “AEC abscissa.” On the contrary, G-75 and G-82 were relatively stable genotypes but had relatively less Fe concentration in their grain (Figure 4A). In the case of grain Zn concentration, it was detected that G-76, G-67, G-68 were the best performers and G-31, followed by G-32, were the least Zn containing genotypes. Among all these Zn rich genotypes, G-76 was the ideal type as this genotype has both high Zn concentration and good stability with less projection in the “AEC ordinate” (Figure 4B). For grain Ca concentration, G-60, G-59, and G-61 were the best performers and G-60 was the ideal genotype. The poor Ca containing genotypes were G-31 followed by G-99 (Figure 4C). Regarding grain protein concentration, the excellent performers were G-64, G-27, G-7, and G-13 while G-71 and G-54 were poor performers. It was detected that G-27 was the ideal genotype considering grain protein concentration (Figure 4D). For grain phytate concentration, a genotype with a low phytate value is desirable to improve the bioavailability of minerals, so genotypes placed opposite to the direction of “AEC abscissa” should be considered. Accordingly, G-99, G-95, G-98, and G-96 were the genotypes with relatively lower values of phytate, and the phytate-rich genotypes were G-75 and G-74. Among the poor performers, G-95 was the ideal genotype and G-99 was the desirable genotype with almost stable performance and having less concentration of phytate in the grain (Figure 4E). Further, based on the five-grain quality traits (Fe, Zn, Ca, protein, and phytate) all the tested genotypes were classified into five major clusters with 18 genotypes in cluster-I, 15 genotypes in cluster-II, 4 genotypes in cluster-III, and 3 genotypes in cluster-IV. G-99 was a unique genotype and the sole occupant of cluster-V, considered the most diverse one (Figure 5).

### Evaluation of Testing Locations

**FIGURE 6:**
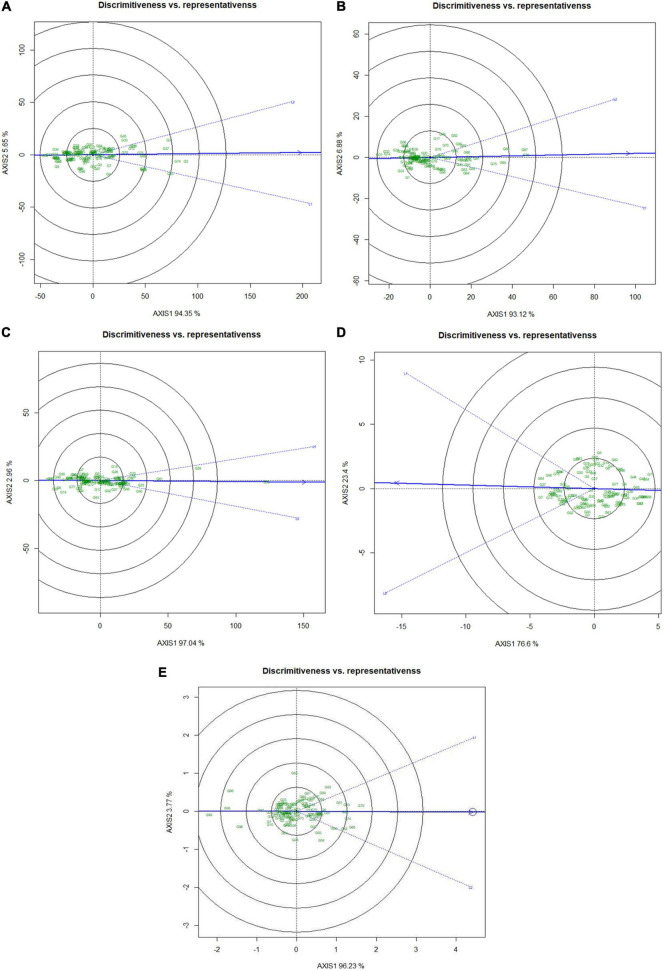
“Discrimitiveness vs. representativeness” view of test locations based on GGE biplot across two testing locations. There was no transformation of data (transform = 0), and data were centered by means of the environments (centering = 2). The biplot was based on ‘row metric preserving.’ Numbers denote serial numbers of genotypes as listed in Supplementary Table S1. **(A)** Discrimitiveness vs. representativeness view of test locations regarding Fe concentration. **(B)** Discrimitiveness vs. representativeness view of test locations regarding Zn concentration. **(C)** Discrimitiveness vs. representativeness view of test locations regarding Ca concentration. **(D)** Discrimitiveness vs. representativeness view of test locations regarding protein concentration. **(E)** Discrimitiveness vs. representativeness view of test locations regarding phytate concentration.

Besides identifying the ideal genotypes, GGE biplot can also detect suitable testing locations for genotypes discrimination based on grain micronutrients and phytate concentration. The relationships among the test locations were enumerated by an environment-centered preserving of data (SPV = 2) without scaling. Regarding the relationship of test locations, it was observed that for Fe, Zn, Ca, and phytate traits both the locations vectors showed acute angle (Figures 6A–C,E). However, in the case of protein concentration, the angles between the two locations were near to obtuse (Figure 6D). Acute vector angles symbolize the closer relationship between the environments and *vice versa*. Therefore, it can be stated that both the locations were highly correlated for all the traits except for protein concentration. The superiority of the test locations in the GGE biplot is measured by the vector length of the test location as “Discriminating ability” on to the target environment. In the case of grain Fe (Figure 6A), Zn (Figure 6B), Ca (Figure 6C), and phytate (Figure 6E), Loc1 was detected as more discriminating than the Loc2 due to having a comparatively longer vector length which indicated that Loc1 was more suitable for genotype discrimination based on their grain phytate, Fe, Zn, and Ca concentration (Table 5). On the contrary, in the case of grain protein concentration it was observed that Loc2 had more discrimination power than the Loc1 (Figure 6D and Table 5). Further, the representativeness of the test locations is denoted by the projection of the environments vectors to the “Average environment axis” (AEA) where locations with acute angles with the AEA are most representative. Accordingly, for most of the traits except for protein concentration, Loc1 was detected as most representative having the power of representing the “mega environment” and closest to the average environment for testing genotypes based on these parameters (Figures 6A–E).

**TABLE 5:** Standardized test location evaluation parameters.

Characters	Discriminating power		Representativeness		Desirability index	
	Loc1	Loc2	Loc1	Loc2	Loc1	Loc2
Fe	9.80	9.39	0.97	0.97	10.8	10.4
Zn	9.75	9.34	0.97	0.96	10.7	10.3
Ca	9.39	9.18	0.89	0.87	10.3	10.1
Protein	9.39	9.60	0.99	0.97	10.4	10.6
Phytate	9.80	9.65	0.98	0.98	10.8	10.6

Additionally, the “Desirability index” of the testing locations was enumerated, which is the cumulative factor of both “discriminating power” and “representativeness” and the conclusive determining factor for detection of suitable testing locations for specific traits. Therefore, Loc1 with high desirability index for Fe (10.8), Zn (10.7), Ca (10.3), and phytate (10.8) was identified as ‘ideal’ or type-I testing location for appraisal of precious genotypes (Table 5). On the contrary, for grain protein concentration, Loc2 (10.6) was detected as an ‘ideal’ location with a better desirability index than Loc1 for better genotypes assessment based on grain protein concentration.

### Correlation Among the Traits

Correlations among the five studied traits in *V. stipulacea* genotypes are presented in Table 6. A significant positive correlation (*r* = 0.34; *p* < 0.01) was detected between grain Fe and Zn concentration considering the pooled analysis over both the locations. Individually, and also in both the locations, grain Fe and Zn concentration revealed significant positive association (data not presented), which implied that both the locations were ideal for screening of *V. stipulacea* genotypes for grain Fe and Zn concentration. The grain protein concentration (*r* = 0.20; *p* < 0.01) also exhibited significant positive association with grain Fe concentration. Additionally, grain phytate and Zn concentration also exhibited positive significant association (*r* = 0.38; *p* < 0.01) over the locations. On the contrary, a significant negative association was observed between phytate and protein concentrations (*r* = 0.27; *p* < 0.01) over the locations. The yield was detected to have a non-significant association with all the grain quality traits.

**TABLE 6:** Correlation analysis among different grain nutrients and antinutrient composition in *V. stipulacea* accessions.

Characters	Fe	Zn	Ca	Protein	Phytate
Zn	0.34**				
Ca	0.02	−0.09			
Protein	0.20*	−0.05	−0.08		
Phytate	0.12	0.38**	0.07	−0.27**	
Yield	0.05	−0.02	0.08	−0.03	0.07

*P < 0.05 and **P < 0.01.

## Discussion

Micronutrient deficiency (MND) or “hidden hunger” is considered a global crisis (Bouis et al., 2013; Wakeel et al., 2018). The basic reason for MNDs in developing countries is the consumption of diets based only on cereals and one or two staples, with less diversification in their food platter due to acute poverty. Pulses are an integral component of the daily diet of vegans as well as the peoples of developing countries. Besides being a chief source of protein, pulses are also a good source of dietary fiber, low molecular weight carbohydrates, essential amino acids, polyunsaturated fatty acids, minerals, and vitamins (Thompson, 2019; Venkidasamy et al., 2019). Hence, pulses are a good candidate for biofortification, and progress of pulse biofortification through conventional breeding programs is also at a good pace (Nair et al., 2013; Petry et al., 2015; Upadhyaya et al., 2016; Kumar et al., 2018; Tan et al., 2018; Jha and Warkentin, 2020; Wu et al., 2020). However, most of the earlier biofortification program targeted major pulses, and the potential genetic diversity of underutilized pulses and crop wild relatives (CWR), which are a rich source of essential elements (Difo et al., 2015), were not harnessed assiduously to unravel the novel alleles governing micronutrient accumulation and their further transfer into the cultivated background. Although *V. stipulacea*, like other *Vigna* species, is nutrient-dense, it received less attention concerning micronutrient composition in the prevention of MNDs, mainly owing to its minor crop status.

This experiment was conducted with the aim of evaluating a large number of genotypes of *V. stipulacea* to understand the presence of variability regarding grain nutrients (Fe, Zn, Ca, and protein) and the antinutritional factor (phytate), followed by contemplating the complex role of environments on the inheritance of these quantitative traits for identifying stable genotypes. In the present study, the mean performance of the accessions revealed the presence of ample genetic variability in most of the studied traits. As a result, the potential to obtain desirable recombinants was emphasized by using promising genotypes as parents in the biofortification efforts. The variability observed in grain nutrients and phytate concentration in the present study mostly corroborated with the variability observed in other *Vigna* species especially for well-studied traits like Fe and Zn (Dwivedi et al., 2012; Nair et al., 2013; Wu et al., 2020). In the present study, the seed Fe concentration of *V. stipulacea* genotypes was 34.3–128.8 mg kg^–1^ over the locations with an average value of 66.7. In earlier studies, with other *Vigna* species like mungbean, an average value of 30.0 and 40.0 mg kg^–1^ of seed Fe have been reported (Wu et al., 2020), which confirmed that *V. stipulacea* is the potential source for mitigating Fe deficiency of rural folks especially adolescent children and female with acute problems of anemia. Similarly, the present finding regarding grain Fe concentration was corroborated with the wider variation reported in the case of other small-seeded legumes like lentil (Thavarajah et al., 2017; Kumar et al., 2018; Shrestha et al., 2018); common bean (de Araújo et al., 2003; Blair et al., 2009) and cowpea (Boukar et al., 2011; Santos and Boiteux, 2015). On the contrary, a narrow range of variation (20.6–71.0 mg kg^–1^) was reported regarding grain Zn concentration compared to grain Fe in our study. In earlier reports with mungbean, a range of 25.0–30.0 mg kg^–1^ of grain Zn was reported (Wu et al., 2020), which further proved that considerably higher Zn concentration was present in *V. stipulacea* genotypes. However, a relatively higher concentration of Zn concentration of 5–134 mg kg^–1^ was reported in urdbean (Gupta et al., 2020). Earlier works in common bean reported a smaller range of seed Zn concentration (Gelin et al., 2007; Blair et al., 2009). The present study revealed that *V. stipulacea* accessions have a greater variation concerning grain Fe concentration than the grain Zn concentration. The same trend was observed in both small and large-seeded grain legumes, perhaps due to changes in embryo size and the proportion of seed coat to cotyledonary tissues (Ariza-Nieto et al., 2007; Cvitanich et al., 2010). In the present study, the differences in Ca concentration also showed very promising results, with an average value ranging between 159.1 and 161.6 mg kg^–1^ across the locations. Differences in the concentration of minerals like Ca are less studied in *Vigna* species, which is highly influenced by the soil pH (Wu et al., 2020). The variation regarding grain protein concentration in the studied *V. stipulacea* accessions was narrow and it was well corroborated with earlier studies in mungbean (Akaerue and Onwuka, 2010; Nair et al., 2013); cowpea (Gupta et al., 2010; Ravelombola et al., 2016) and urdbean (Shaheen et al., 2012). Phytate or phytic acid is considered as one of the antinutritional factors and inhibits the bioavailability of minerals and proteins (Kumar et al., 2010; Nissar et al., 2017). Paradoxically, phytate also has nutraceutical properties and is considered a useful suppressor of cardiovascular diseases and cancers in human beings (Selle et al., 2012; Thavarajah et al., 2014). Besides, phytate is an essential component for plant metabolism (Oomah et al., 2011). Therefore, biofortification efforts for reducing phytate concentration should be considered meticulously so that an optimum balance between seed phytate concentration and bioavailability of other minerals can be judiciously accomplished. An earlier study by Chitra et al. (1995) reported that phytate concentration was the highest in urdbean followed by mungbean with a concentration of 13.7 mg g^–1^ and 12.0 mg g^–1^, respectively. The phytate concentration in *V. stipulacea* accessions was higher (15.4 mg g^–1^) than both urdbean and mungbean, which requires urgency to initiate a research program for the reduction of the phytate concentration of this *Vigna* species to an optimum level so that the bioavailability of the minerals can be improved. Although, the phytate concentration of *V. stipulacea* was comparatively lesser than legumes such as soybean (mg g^–1^), as mentioned by Chitra et al. (1995). Furthermore, if the heritability for the characteristic of interest is high, the variability existing in the population is helpful and can be harnessed. In the present study, high heritability coupled with high GA for most of the studied traits justified that any selection will be rewarding for improving these traits in *V. stipulacea*.

The perplexing role of the environment and the networking between GEI is a serious noise toward the phenotypic expression of any quantitative traits due to a reduction in heritability (Phuke et al., 2017). Therefore, GEI should be judged properly by growing the genotypes in different locations to confirm the stable phenotypic expression regarding complex traits of interest. GEI is the product of different kinds of genetic association across the environments (Ye et al., 2006). In the present study, the ANOVA for all the studied traits represented highly significant genotypic variance across the environments based on pooled analysis. Similarly, the interplay between genotype and location interactions (σ^2^gl) was also highly significant for individual context, suggesting the predominant role of genotype, environment, and their interaction (GEI) toward the inheritance of these traits. This finding justified the partitioning of GEI interaction. Grain micronutrients concentration is highly variable on environmental factors, especially soil properties of the location (Bashir et al., 2014; Kumar et al., 2018), which hinders the progress of genetic analysis of these traits. It was observed that the genotype × location (σ^2^gl) was the highest for phytate concentration due to higher genotypic variance for this trait in *V. stipulacea* genotypes. Moreover, high σ^2^gl was also observed in grain Fe concentration which was corroborated with the findings of Gupta et al. (2020) in urdbean; mungbean (Wu et al., 2020), and cowpea (Boukar et al., 2011; Santos and Boiteux, 2015).

In a crop biofortification program with the objective of improving the nutritional quality of food crops, determining the environmental stability concerning grain micronutrients is imperative (Welch and Graham, 2004; Upadhyaya et al., 2012; Bouis and Saltzman, 2017). GEI impedes genotypic selection based on phenotypic expression and ultimately reduces the genetic gain under selection (Comstock and Moll, 1963). Despite these challenges of GEI in crop biofortification program, breeders were enabled to develop nutrient dense biofortified varieties in many food legumes through exploiting various methodologies for stable genotype delineation (Boukar et al., 2011; Upadhyaya et al., 2012; Santos and Boiteux, 2015; Kumar et al., 2018). Recently among the various statistical tools for determining GEI, GGE biplot is gaining attention to analyze multi-locational data for deciphering complex GEI in a graphical mode (Yan, 2001; Yan and Tinker, 2006; Yan et al., 2007). In GGE biplot analysis, the complex GEI are presented in the form of various PCs for graphical presentation of the data against each PC (Yan and Tinker, 2006). Earlier findings stated that the first two PCs should explain more than 60.0% of the variability present within the data set for determining the competence of the methodology (Yan et al., 2010). In the present study, both the PCs were able to explain the total variations for all the studied traits. For detection of the ideal genotype, both the mean performance and consistency over the locations should be considered (Yan et al., 2007). The presence of high GEI for all the studied traits influenced the rank of the genotypes across the locations, suggesting the presence of COI as reported earlier (Das et al., 2019; Singh B. et al., 2020). The presence of COI recommended breeding for specific adaptation.

In the present dataset, it was detected that the highest grain Fe (G-2), Zn (G-76), Ca (G-60), and protein (G-64) containing genotypes were not stable except in the case of grain Ca concentration where G-60 was the most stable as well as a high performer thus considered as the ‘ideal’ genotype. For grain Fe, Zn, and protein concentration, the ‘ideal’ genotype were G-75, G-76, and G-27, respectively. For phytate concentration, the low performing genotypes (G-99, G-95) exhibited stable performance and were acknowledged as ‘ideal’ (G-95) and ‘desirable’ (G-99). Genotypes which positioned close to the ‘ideal’ genotype are considered ‘desirable’ as the distance between two genotypes resembled the Euclidian distance between them (Yan and Tinker, 2006). Among all the tested traits except for grain Ca, COI was observed for all the traits. Therefore, it can be said that within the same data set, both COI and non-COI were detected which was in affirmation with earlier findings (Dehghani et al., 2006; Sabaghnia et al., 2008; Das et al., 2019, 2020; Singh B. et al., 2020). The identified genotypes (G-75, G-76, G-60, G-27, G-95) for all the five studied traits would be precious genetic stocks for utilization as parents in a *V. stipulacea* biofortification program.

Besides identification of stable genotypes, delineation of the best testing location is also the choice of breeders. In GGE biplot, an ‘ideal’ test environment should be selected based on “discriminating power” to delineate genotypes, being ‘representative’ as well as having a high “desirability index” (Xu et al., 2014). In the present study, Loc1 was considered as the ‘ideal’ testing location for most of the traits except grain protein, where Loc2 was detected as the most ‘ideal.’ Ideal testing location delimitation would facilitate plant breeders for conducting their trials meticulously for precise genotype selection. Previous studies applied the same principle of GGE for genotype and testing location appraisal in different legumes concerning grain micronutrient concentration (Janila et al., 2015; Phuke et al., 2017; Darai et al., 2020; Gupta et al., 2020; Misra et al., 2020).

The association between different grain micronutrients stated that grain Fe and Zn exhibited a significant positive association that followed the same trend in individual locations. Similar relationships between these micronutrients have been reported in earlier studies in urdbean (Gupta et al., 2020); peanut (Janila et al., 2015); and chickpea (Misra et al., 2020). It can be speculated that the positive association of grain Fe and Zn might be due to overlapping QTLs as reported earlier in common bean (Blair et al., 2009, 2010), suggesting simultaneous selection for both the traits would be effective. Similarly, grain protein exhibited a significant positive association with Fe, which was corroborated with the earlier finding in wheat (Cakmak et al., 2010) and pearl millet (Pujar et al., 2020). On the contrary, a significant negative correlation between grain Fe and protein was detected in cowpea (Gerrano et al., 2019). The present study detected a significant negative association between grain protein and phytate. However, earlier studies reported a positive association between these two traits (Chitra et al., 1995; Dai et al., 2007), though the amplitude of correlation was relatively modest in the case of pulses. Therefore, selection against phytate concentration would be rewarding toward the selection of high protein genotypes in *V. stipulacea*.

## Conclusion

The adequate variability was observed regarding grain nutrients and phytate concentration among the *V. stipulacea* germplasm. The presence of significant GEI for most of the traits indicated the convoluted role of environments on the phenotypic expression of these traits. A high magnitude of GEI was observed for grain phytate and Fe concentration. GGE biplot analysis revealed the incongruous performance of the genotypes for most of the traits except grain Ca concentration, suggesting precise phenotyping of the traits in a testing location with specific adaptation. GGE biplot methodology deployed in the present study decisively delineated stable genotypes concerning grain nutrients and phytate as well as testing locations for culling out ideal genotypes. The positive and significant association between grain Fe with Zn and protein admitted the possibilities of simultaneous selection of all these three characters. Commonly, most of the *Vigna* species are native to Asia and the horn of Africa and are prevalent in the food platter of Asian and African communities where MNDs are very acute. Like other *Vigna* species, *V*. *stipulacea* is also easy to cook quickly; therefore, it requires meager energy demands during food preparation. Wider variation of essential nutrients in *V. stipulacea* followed by utilization of stable genotypes in a biofortification program holds immense promise to combat the malnutrition of Sub-Saharan Africa and other underdeveloped countries of Asia and Africa in a judicious way.

## Data Availability Statement

The original contributions presented in the study are included in the article/Supplementary Material, further inquiries can be directed to the corresponding authors.

## Author Contributions

PG and KT conceptualized the experiments. PG performed the experiments and prepared the manuscript. AD, KT, and SB analyzed the data and prepared the manuscript. AP contributed to the facilitation of the field trials and data recording. RB provided lab facility for biochemical analysis. VG and HD supervised the research trial. RN contributed to the acquisition of fund, revision and finalization of the manuscript. All authors have read and agreed to the final version of the manuscript.

## Conflict of Interest

The authors declare that the research was conducted in the absence of any commercial or financial relationships that could be construed as a potential conflict of interest.

## Publisher’s Note

All claims expressed in this article are solely those of the authors and do not necessarily represent those of their affiliated organizations, or those of the publisher, the editors and the reviewers. Any product that may be evaluated in this article, or claim that may be made by its manufacturer, is not guaranteed or endorsed by the publisher.
