# Supplementary figures and images for: Understanding G × E Interaction for Nutritional and Antinutritional Factors in a Diverse Panel of Vigna stipulacea (Lam.) Kuntz Germplasm Tested Over the Locations
Source: Front Plant Sci. 2021 Dec 13;12:766645. doi: 10.3389/fpls.2021.766645 (PMC8710513; doi:10.3389/fpls.2021.766645)

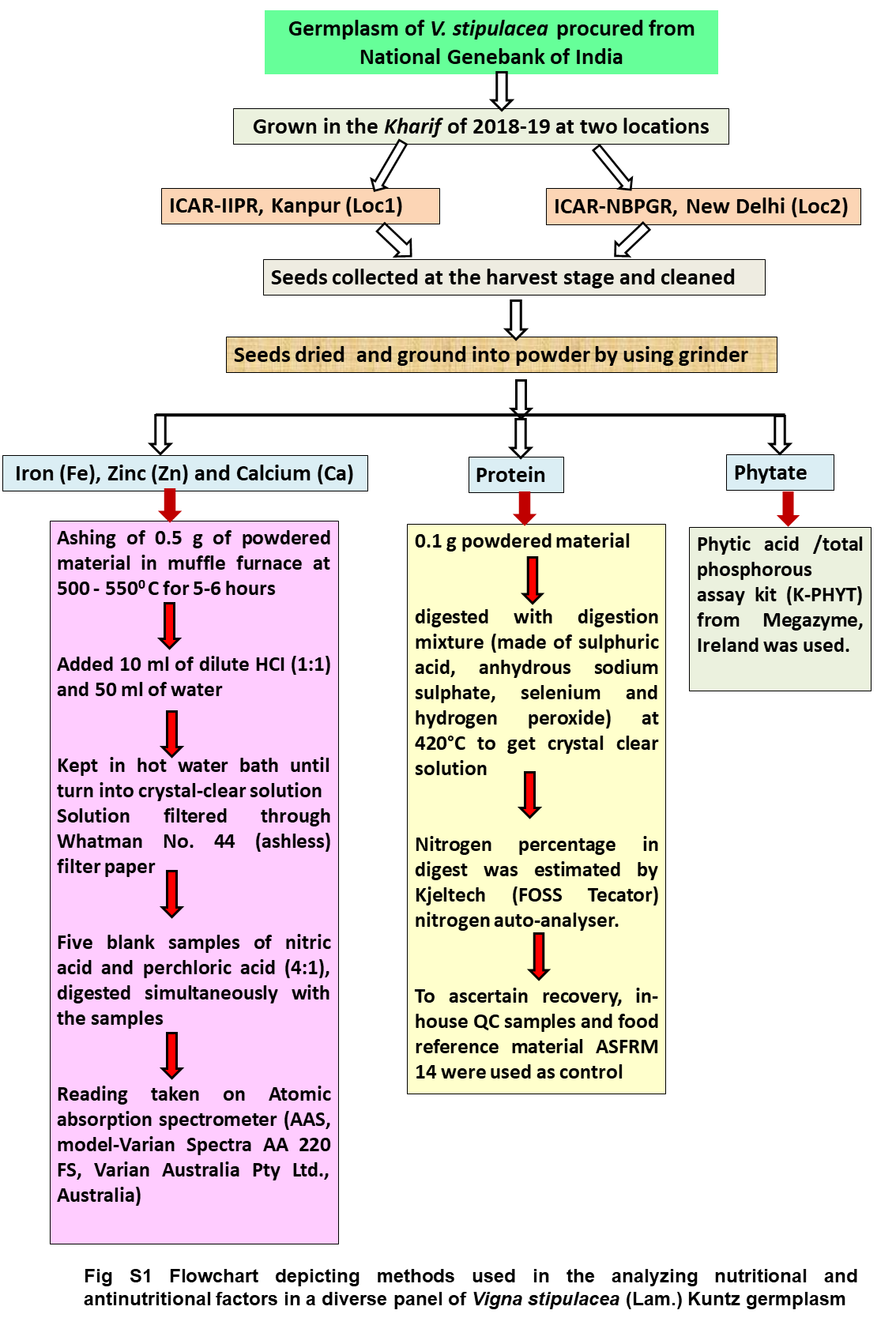

Supplement: Supplementary file 1 [file Image_1.tif]
