# Supplementary material for: Understanding G × E Interaction for Nutritional and Antinutritional Factors in a Diverse Panel of Vigna stipulacea (Lam.) Kuntz Germplasm Tested Over the Locations
Source: Front Plant Sci. 2021 Dec 13;12:766645. doi: 10.3389/fpls.2021.766645 (PMC8710513; doi:10.3389/fpls.2021.766645)
Supplement: Supplementary file 2 [file Table_1.DOCX]

**Table S1 Grain yield and grain concentrations of Fe, Zn, Ca, protein and phytate in 99 *Vigna stipulacea* genotypes grown in two locations.**

| **Accession no** | **Genotypes** | **Fe (mg kg^-1^)** | | | **Zn (mg kg^-1^)** | | | **Ca(mg kg^-1^)** | | | **Protein (%)** | | | **Phytate (mg g^-1^)** | | | **Grain yield (g plant^-1^)** | | |
| --- | --- | --- | --- | --- | --- | --- | --- | --- | --- | --- | --- | --- | --- | --- | --- | --- | --- | --- | --- |
|  |  | **Loc1** | **Loc2** | **Mean** | **Loc1** | **Loc2** | **Mean** | **Loc1** | **Loc2** | **Mean** | **Loc1** | **Loc2** | **Mean** | **Loc1** | **Loc2** | **Mean** | **Loc1** | **Loc2** | **Mean** |
| IC252016 | G1 | 62.3 | 60.0 | 61.2 | 37.1 | 22.2 | 29.7 | 155 | 153 | 154 | 22.9 | 23.6 | 23.3 | 12.4 | 12.1 | 12.3 | 43.4 | 39.5 | 41.4 |
| IC261321 | G2 | 131 | 127 | 129 | 35.4 | 26.7 | 31.1 | 161 | 156 | 159 | 22.4 | 24.3 | 23.4 | 13.9 | 13.5 | 13.7 | 31.4 | 38.5 | 34.9 |
| IC261384 | G3 | 106 | 130 | 118 | 35.4 | 26.2 | 30.8 | 158 | 156 | 157 | 20.4 | 23.9 | 22.2 | 13.9 | 12.3 | 13.1 | 46.0 | 54.9 | 50.5 |
| IC305192 | G4 | 86.1 | 67.4 | 76.7 | 33.2 | 28.4 | 30.8 | 148 | 152 | 150 | 20.3 | 20.1 | 20.2 | 15.0 | 14.5 | 14.7 | 51.6 | 40.4 | 46.0 |
| IC553494 | G5 | 73.5 | 68.2 | 70.9 | 34.4 | 26.3 | 30.3 | 162 | 160 | 161 | 21.7 | 22.4 | 22.0 | 14.4 | 13.2 | 13.8 | 50.1 | 49.1 | 49.6 |
| IC610275 | G6 | 62.3 | 52.8 | 57.6 | 32.8 | 31.0 | 31.9 | 136 | 142 | 139 | 22.1 | 20.3 | 21.2 | 19.2 | 17.6 | 18.4 | 86.4 | 74.4 | 80.4 |
| IC524667 | G7 | 80.8 | 73.5 | 77.1 | 29.5 | 33.9 | 31.7 | 157 | 143 | 150 | 23.8 | 25.7 | 24.8 | 14.2 | 13.5 | 13.8 | 45.0 | 33.9 | 39.5 |
| IC550531 | G8 | 44.6 | 39.1 | 41.9 | 33.4 | 29.1 | 31.3 | 164 | 153 | 159 | 22.7 | 21.9 | 22.3 | 14.1 | 12.7 | 13.4 | 36.1 | 35.2 | 35.7 |
| IC550532 | G9 | 49.4 | 61.8 | 55.6 | 32.3 | 25.9 | 29.1 | 166 | 167 | 167 | 23.5 | 20.0 | 21.7 | 15.4 | 13.6 | 14.5 | 64.3 | 59.1 | 61.7 |
| IC550533 | G10 | 38.0 | 44.9 | 41.5 | 33.7 | 31.5 | 32.6 | 170 | 169 | 169 | 21.4 | 23.9 | 22.6 | 12.4 | 12.4 | 12.4 | 57.9 | 47.5 | 52.7 |
| IC550536 | G11 | 54.2 | 65.2 | 59.7 | 36.0 | 34.3 | 35.2 | 174 | 167 | 170 | 23.2 | 20.5 | 21.9 | 14.7 | 13.6 | 14.1 | 62.1 | 51.1 | 56.6 |
| IC550538 | G12 | 85.3 | 100 | 92.7 | 33.2 | 29.5 | 31.4 | 169 | 169 | 169 | 22.5 | 24.1 | 23.3 | 14.7 | 13.4 | 14.0 | 57.4 | 51.5 | 54.5 |
| IC550545 | G13 | 68.4 | 82.9 | 75.7 | 33.2 | 28.0 | 30.6 | 170 | 170 | 170 | 23.4 | 25.2 | 24.3 | 13.6 | 12.7 | 13.2 | 45.4 | 54.2 | 49.8 |
| IC550548 | G14 | 38.8 | 44.8 | 41.8 | 22.1 | 24.4 | 23.3 | 136 | 147 | 141 | 22.5 | 20.1 | 21.3 | 15.4 | 14.2 | 14.8 | 24.1 | 33.1 | 28.6 |
| IC550551 | G15 | 62.5 | 76.4 | 69.4 | 37.2 | 32.2 | 34.7 | 177 | 158 | 167 | 24.4 | 23.5 | 24.0 | 14.8 | 12.5 | 13.6 | 64.6 | 73.0 | 68.8 |
| IC550553 | G16 | 68.2 | 84.1 | 76.1 | 41.7 | 36.7 | 39.2 | 179 | 173 | 176 | 24.4 | 23.3 | 23.9 | 13.7 | 13.0 | 13.3 | 67.2 | 61.6 | 64.4 |
| IC553502 | G17 | 59.8 | 68.0 | 63.9 | 35.4 | 45.8 | 40.6 | 156 | 162 | 159 | 22.2 | 21.6 | 21.9 | 14.6 | 12.5 | 13.5 | 51.8 | 41.5 | 46.6 |
| IC524639 | G18 | 85.2 | 103 | 94.3 | 30.7 | 26.7 | 28.7 | 159 | 158 | 159 | 23.5 | 24.8 | 24.1 | 15.2 | 13.6 | 14.4 | 22.1 | 18.2 | 20.1 |
| IC553505 | G19 | 59.7 | 73.3 | 66.5 | 32.8 | 31.7 | 32.3 | 162 | 162 | 162 | 23.5 | 21.2 | 22.4 | 14.2 | 14.3 | 14.2 | 49.6 | 47.0 | 48.3 |
| IC553509 | G20 | 52.2 | 70.6 | 61.4 | 35.6 | 36.7 | 36.1 | 151 | 148 | 150 | 23.3 | 22.1 | 22.7 | 16.1 | 13.9 | 15.0 | 49.1 | 36.2 | 42.7 |
| IC553510 | G21 | 41.6 | 54.3 | 47.9 | 28.4 | 28.0 | 28.2 | 155 | 146 | 151 | 23.1 | 24.6 | 23.8 | 14.7 | 12.6 | 13.6 | 43.5 | 41.1 | 42.3 |
| IC553512 | G22 | 60.7 | 64.2 | 62.5 | 39.5 | 34.0 | 36.8 | 183 | 171 | 177 | 23.4 | 20.4 | 21.9 | 15.0 | 13.8 | 14.4 | 58.5 | 51.5 | 55.0 |
| IC553516 | G23 | 84.5 | 102 | 93.4 | 38.6 | 32.7 | 35.7 | 182 | 181 | 182 | 22.9 | 21.5 | 22.2 | 15.1 | 12.5 | 13.8 | 69.8 | 61.7 | 65.7 |
| IC553517 | G24 | 44.6 | 66.8 | 55.7 | 33.5 | 32.7 | 33.1 | 166 | 161 | 164 | 22.4 | 23.8 | 23.1 | 13.6 | 14.4 | 14.0 | 62.3 | 69.1 | 65.7 |
| IC553518 | G25 | 47.2 | 57.8 | 52.5 | 38.8 | 34.7 | 36.8 | 172 | 171 | 171 | 22.1 | 19.9 | 21.0 | 14.4 | 16.0 | 15.2 | 59.8 | 53.3 | 56.6 |
| IC553520 | G26 | 70.8 | 85.7 | 78.3 | 34.2 | 33.0 | 33.6 | 174 | 161 | 168 | 22.3 | 23.6 | 23.0 | 14.6 | 14.3 | 14.4 | 62.4 | 55.7 | 59.0 |
| IC553521 | G27 | 57.2 | 75.3 | 66.2 | 34.3 | 31.7 | 33.0 | 175 | 171 | 173 | 24.3 | 24.9 | 24.6 | 14.5 | 12.6 | 13.5 | 62.5 | 60.2 | 61.4 |
| IC553522 | G28 | 46.3 | 64.6 | 55.5 | 32.2 | 30.2 | 31.2 | 172 | 167 | 169 | 19.4 | 20.8 | 20.1 | 13.0 | 11.7 | 12.3 | 59.9 | 48.4 | 54.1 |
| IC553523 | G29 | 37.8 | 44.7 | 41.3 | 28.6 | 31.4 | 30.0 | 181 | 173 | 177 | 23.5 | 23.7 | 23.6 | 15.1 | 14.2 | 14.7 | 69.4 | 61.3 | 65.3 |
| IC553524 | G30 | 42.9 | 56.7 | 49.8 | 30.9 | 35.1 | 33.0 | 174 | 169 | 171 | 23.2 | 21.5 | 22.4 | 15.6 | 13.7 | 14.6 | 54.2 | 49.7 | 51.9 |
| IC553525 | G31 | 37.8 | 45.3 | 41.6 | 19.4 | 21.7 | 20.6 | 135 | 133 | 134 | 22.9 | 20.6 | 21.8 | 14.6 | 13.7 | 14.1 | 22.8 | 29.6 | 26.2 |
| IC553526 | G32 | 31.3 | 42.8 | 37.0 | 21.0 | 25.1 | 23.1 | 174 | 171 | 173 | 22.6 | 20.5 | 21.6 | 16.1 | 14.0 | 15.1 | 39.5 | 31.4 | 35.4 |
| IC553534 | G33 | 75.1 | 102 | 88.5 | 32.5 | 22.5 | 27.5 | 171 | 170 | 170 | 20.5 | 21.8 | 21.2 | 14.4 | 12.3 | 13.4 | 58.8 | 47.8 | 53.3 |
| IC553535 | G34 | 32.5 | 50.7 | 41.6 | 28.3 | 31.9 | 30.1 | 171 | 172 | 171 | 18.9 | 20.2 | 19.6 | 12.9 | 12.1 | 12.5 | 59.0 | 46.5 | 52.7 |
| IC553527 | G35 | 45.8 | 64.9 | 55.4 | 24.2 | 28.7 | 26.5 | 162 | 159 | 161 | 21.3 | 22.7 | 22.0 | 15.2 | 14.5 | 14.9 | 49.6 | 37.2 | 43.4 |
| IC553528 | G36 | 61.0 | 73.0 | 67.0 | 26.2 | 29.6 | 27.9 | 165 | 164 | 164 | 21.5 | 23.2 | 22.4 | 14.9 | 15.0 | 14.9 | 52.8 | 41.2 | 47.0 |
| IC553529 | G37 | 110 | 122 | 116 | 35.1 | 31.3 | 33.2 | 174 | 172 | 173 | 23.3 | 21.3 | 22.3 | 14.1 | 12.8 | 13.5 | 41.9 | 47.7 | 44.8 |
| IC553530 | G38 | 64.3 | 81.3 | 72.8 | 34.8 | 32.6 | 33.7 | 172 | 166 | 169 | 22.4 | 24.8 | 23.6 | 14.2 | 13.2 | 13.7 | 60.0 | 48.5 | 54.3 |
| IC553531 | G39 | 41.9 | 55.4 | 48.7 | 34.1 | 31.6 | 32.9 | 175 | 174 | 174 | 19.6 | 20.2 | 19.9 | 14.4 | 12.7 | 13.5 | 62.9 | 50.4 | 56.7 |
| IC553532 | G40 | 60.9 | 65.6 | 63.3 | 30.8 | 32.7 | 31.8 | 178 | 184 | 181 | 20.6 | 22.4 | 21.5 | 14.5 | 13.2 | 13.9 | 18.6 | 21.3 | 19.9 |
| IC553537 | G41 | 53.4 | 73.1 | 63.3 | 28.5 | 31.3 | 29.9 | 173 | 172 | 173 | 18.6 | 19.8 | 19.2 | 15.4 | 13.3 | 14.4 | 60.8 | 50.4 | 55.6 |
| IC553538 | G42 | 39.8 | 45.1 | 42.4 | 30.9 | 30.3 | 30.6 | 151 | 152 | 152 | 19.7 | 20.7 | 20.2 | 14.3 | 14.3 | 14.3 | 61.7 | 62.9 | 62.3 |
| IC553539 | G43 | 49.5 | 60.5 | 55.0 | 28.9 | 27.9 | 28.4 | 155 | 153 | 154 | 19.7 | 18.9 | 19.3 | 15.4 | 14.9 | 15.1 | 42.6 | 39.9 | 41.2 |
| IC553540 | G44 | 42.5 | 43.9 | 43.2 | 34.2 | 31.2 | 32.7 | 143 | 141 | 142 | 24.4 | 23.2 | 23.8 | 17.6 | 14.9 | 16.3 | 32.7 | 24.8 | 28.8 |
| IC553541 | G45 | 71.5 | 104 | 87.5 | 47.4 | 36.4 | 41.9 | 155 | 153 | 154 | 19.5 | 19.6 | 19.6 | 18.1 | 16.0 | 17.0 | 61.4 | 52.8 | 57.1 |
| IC553544 | G46 | 40.4 | 54.9 | 47.7 | 33.6 | 31.7 | 32.7 | 155 | 149 | 152 | 20.3 | 19.3 | 19.8 | 17.1 | 16.2 | 16.6 | 30.9 | 25.7 | 28.3 |
| IC553547 | G47 | 42.3 | 55.5 | 48.9 | 32.2 | 30.9 | 31.5 | 154 | 149 | 151 | 20.5 | 21.3 | 20.9 | 17.3 | 15.2 | 16.3 | 42.0 | 39.0 | 40.5 |
| IC553548 | G48 | 48.3 | 63.3 | 55.8 | 37.2 | 35.9 | 36.5 | 154 | 153 | 154 | 20.8 | 21.5 | 21.1 | 17.4 | 16.8 | 17.1 | 42.2 | 39.4 | 40.8 |
| IC553551 | G49 | 68.6 | 80.1 | 74.4 | 52.2 | 43.6 | 47.9 | 157 | 148 | 152 | 23.3 | 24.3 | 23.8 | 14.9 | 13.6 | 14.2 | 45.1 | 39.1 | 42.1 |
| IC550520 | G50 | 44.8 | 56.9 | 50.8 | 34.2 | 32.8 | 33.5 | 172 | 166 | 169 | 21.8 | 23.4 | 22.6 | 13.6 | 12.4 | 13.0 | 60.2 | 53.8 | 57.0 |
| IC553553 | G51 | 42.3 | 44.5 | 43.4 | 38.9 | 35.0 | 37.0 | 162 | 161 | 161 | 19.6 | 20.6 | 20.1 | 21.3 | 18.9 | 20.1 | 59.8 | 47.4 | 53.6 |
| IC553554 | G52 | 50.9 | 54.3 | 52.6 | 36.4 | 31.8 | 34.1 | 161 | 158 | 160 | 20.0 | 22.3 | 21.1 | 20.8 | 16.8 | 18.8 | 49.5 | 41.5 | 45.5 |
| IC553555 | G53 | 68.4 | 75.9 | 72.2 | 25.7 | 33.0 | 29.4 | 164 | 164 | 164 | 19.4 | 21.6 | 20.5 | 17.7 | 12.4 | 15.0 | 62.1 | 50.2 | 56.2 |
| IC553556 | G54 | 53.2 | 76.0 | 64.6 | 34.0 | 31.4 | 32.7 | 164 | 157 | 161 | 19.5 | 18.6 | 19.1 | 17.4 | 18.9 | 18.1 | 61.2 | 55.3 | 58.2 |
| IC553557 | G55 | 42.3 | 53.4 | 47.9 | 32.3 | 31.8 | 32.0 | 152 | 149 | 151 | 20.2 | 22.4 | 21.3 | 14.2 | 12.4 | 13.3 | 40.3 | 37.6 | 39.0 |
| IC553558 | G56 | 29.4 | 39.3 | 34.3 | 24.1 | 33.9 | 29.0 | 153 | 147 | 150 | 21.2 | 19.6 | 20.4 | 15.0 | 13.9 | 14.5 | 41.0 | 33.0 | 37.0 |
| IC553560 | G57 | 44.4 | 56.6 | 50.5 | 33.9 | 33.2 | 33.5 | 154 | 152 | 153 | 24.2 | 21.6 | 22.9 | 17.5 | 15.9 | 16.7 | 41.6 | 32.9 | 37.3 |
| IC553561 | G58 | 35.5 | 46.8 | 41.2 | 26.2 | 33.2 | 29.7 | 153 | 148 | 151 | 20.7 | 23.1 | 21.9 | 18.5 | 17.2 | 17.9 | 49.5 | 38.8 | 44.2 |
| IC553562 | G59 | 43.6 | 53.7 | 48.7 | 38.2 | 34.4 | 36.3 | 221 | 201 | 211 | 21.4 | 24.1 | 22.8 | 18.4 | 15.6 | 17.0 | 23.0 | 18.6 | 20.8 |
| IC553564 | G60 | 77.0 | 80.9 | 78.9 | 40.9 | 38.0 | 39.5 | 252 | 243 | 247 | 20.3 | 21.5 | 20.9 | 17.5 | 14.5 | 16.0 | 23.0 | 16.7 | 19.8 |
| IC553565 | G61 | 60.7 | 62.0 | 61.3 | 41.3 | 33.7 | 37.5 | 195 | 187 | 191 | 19.5 | 22.5 | 21.0 | 18.2 | 14.9 | 16.6 | 18.9 | 21.6 | 20.3 |
| IC622860 | G62 | 62.0 | 62.1 | 62.0 | 40.9 | 39.2 | 40.1 | 156 | 152 | 154 | 22.2 | 19.4 | 20.8 | 20.5 | 20.8 | 20.6 | 43.9 | 49.8 | 46.9 |
| IC622861 | G63 | 129 | 107 | 118 | 55.4 | 43.2 | 49.3 | 154 | 152 | 153 | 24.2 | 21.4 | 22.8 | 22.0 | 19.8 | 20.9 | 75.3 | 67.3 | 71.3 |
| IC276983 | G64 | 61.3 | 81.2 | 71.2 | 57.4 | 42.5 | 50.0 | 156 | 154 | 155 | 25.0 | 24.4 | 24.7 | 16.1 | 14.8 | 15.5 | 44.0 | 41.9 | 43.0 |
| IC622865 | G65 | 51.5 | 67.0 | 59.2 | 67.5 | 58.2 | 62.9 | 165 | 162 | 163 | 21.4 | 19.6 | 20.5 | 21.5 | 21.6 | 21.5 | 52.7 | 49.9 | 51.3 |
| IC210580 | G66 | 60.9 | 73.9 | 67.4 | 51.0 | 50.3 | 50.7 | 165 | 166 | 166 | 19.6 | 21.4 | 20.5 | 17.1 | 14.5 | 15.8 | 39.8 | 37.3 | 38.5 |
| IC251435 | G67 | 49.2 | 55.2 | 52.2 | 71.4 | 69.8 | 70.6 | 151 | 143 | 147 | 21.4 | 23.2 | 22.3 | 16.8 | 14.4 | 15.6 | 39.0 | 36.1 | 37.5 |
| IC024837 | G68 | 76.5 | 83.1 | 79.8 | 64.3 | 64.2 | 64.3 | 152 | 146 | 149 | 20.3 | 21.6 | 21.0 | 15.1 | 12.9 | 14.0 | 53.2 | 47.5 | 50.4 |
| IC251436 | G69 | 74.0 | 77.6 | 75.8 | 47.5 | 51.7 | 49.6 | 161 | 160 | 161 | 20.6 | 22.4 | 21.5 | 18.3 | 17.1 | 17.7 | 49.1 | 41.2 | 45.2 |
| IC331436 | G70 | 83.5 | 93.2 | 88.4 | 40.8 | 46.2 | 43.5 | 146 | 143 | 144 | 20.6 | 21.7 | 21.2 | 18.1 | 16.5 | 17.3 | 34.1 | 30.4 | 32.2 |
| IC331437 | G71 | 53.3 | 61.5 | 57.4 | 42.4 | 41.2 | 41.8 | 161 | 160 | 161 | 19.5 | 18.1 | 18.8 | 16.2 | 14.5 | 15.4 | 49.2 | 42.4 | 45.8 |
| IC331453 | G72 | 71.3 | 87.4 | 79.3 | 45.8 | 41.9 | 43.8 | 155 | 156 | 156 | 22.7 | 24.6 | 23.7 | 18.5 | 17.4 | 18.0 | 42.6 | 34.3 | 38.5 |
| IC331454 | G73 | 61.4 | 71.9 | 66.6 | 45.6 | 42.4 | 44.0 | 146 | 142 | 144 | 19.6 | 23.2 | 21.4 | 16.1 | 15.4 | 15.8 | 34.2 | 30.5 | 32.4 |
| IC331456 | G74 | 126 | 121 | 123 | 54.2 | 48.0 | 51.1 | 151 | 152 | 152 | 22.5 | 24.6 | 23.5 | 21.4 | 20.7 | 21.1 | 38.8 | 32.0 | 35.4 |
| IC331457 | G75 | 94.4 | 103 | 98.5 | 64.5 | 54.7 | 59.6 | 153 | 152 | 153 | 19.2 | 21.3 | 20.3 | 23.6 | 21.4 | 22.5 | 44.5 | 34.7 | 39.6 |
| IC331610 | G76 | 107 | 93.3 | 100 | 74.2 | 67.8 | 71.0 | 153 | 149 | 151 | 18.7 | 19.8 | 19.3 | 17.4 | 15.1 | 16.2 | 54.2 | 47.8 | 51.0 |
| IC251438 | G77 | 107 | 93.5 | 100 | 53.4 | 48.0 | 50.7 | 143 | 148 | 146 | 20.8 | 20.6 | 20.7 | 17.5 | 16.3 | 16.9 | 31.3 | 27.9 | 29.6 |
| IC349701 | G78 | 69.1 | 71.9 | 70.5 | 39.3 | 42.4 | 40.9 | 170 | 173 | 172 | 19.4 | 21.4 | 20.4 | 17.4 | 15.5 | 16.5 | 19.1 | 22.4 | 20.7 |
| IC351406 | G79 | 67.5 | 73.2 | 70.4 | 36.3 | 49.3 | 42.8 | 161 | 155 | 158 | 23.5 | 24.3 | 23.9 | 17.6 | 16.5 | 17.0 | 74.8 | 64.1 | 69.5 |
| IC417392 | G80 | 75.8 | 81.9 | 78.9 | 45.3 | 50.8 | 48.1 | 172 | 171 | 172 | 21.2 | 23.4 | 22.3 | 18.0 | 17.1 | 17.6 | 43.5 | 41.2 | 42.3 |
| IC622867 | G81 | 51.5 | 61.9 | 56.7 | 56.4 | 51.2 | 53.8 | 164 | 161 | 163 | 20.9 | 23.4 | 22.1 | 19.4 | 18.1 | 18.8 | 52.2 | 40.3 | 46.2 |
| IC622868 | G82 | 52.1 | 64.5 | 58.3 | 41.1 | 52.6 | 46.9 | 166 | 163 | 164 | 20.2 | 21.1 | 20.6 | 21.5 | 20.0 | 20.7 | 53.8 | 42.0 | 47.9 |
| IC622869 | G83 | 48.3 | 51.9 | 50.1 | 50.7 | 45.6 | 48.2 | 165 | 163 | 164 | 20.6 | 23.7 | 22.2 | 15.8 | 14.3 | 15.1 | 53.3 | 47.6 | 50.4 |
| IC521211 | G84 | 57.2 | 61.9 | 59.5 | 31.5 | 32.8 | 32.2 | 149 | 145 | 147 | 21.2 | 23.8 | 22.5 | 19.5 | 16.7 | 18.1 | 37.1 | 34.1 | 35.6 |
| IC521245 | G85 | 60.9 | 71.5 | 66.2 | 37.6 | 33.1 | 35.3 | 152 | 153 | 153 | 20.2 | 21.2 | 20.7 | 17.5 | 15.6 | 16.5 | 40.5 | 29.0 | 34.8 |
| IC521215 | G86 | 49.4 | 53.3 | 51.4 | 42.5 | 34.1 | 38.3 | 153 | 146 | 150 | 18.5 | 19.7 | 19.1 | 14.8 | 14.1 | 14.4 | 40.8 | 31.3 | 36.0 |
| IC259512 | G87 | 74.3 | 63.9 | 69.1 | 53.3 | 49.2 | 51.2 | 165 | 171 | 168 | 18.4 | 20.2 | 19.3 | 18.2 | 15.6 | 16.9 | 53.3 | 49.8 | 51.5 |
| IC037804 | G88 | 49.3 | 51.9 | 50.6 | 43.2 | 40.8 | 42.0 | 172 | 176 | 174 | 18.3 | 19.4 | 18.9 | 18.4 | 17.1 | 17.7 | 60.0 | 55.9 | 58.0 |
| IC622870 | G89 | 51.2 | 63.6 | 57.4 | 58.1 | 46.1 | 52.1 | 146 | 153 | 150 | 21.4 | 23.5 | 22.5 | 19.9 | 16.5 | 18.2 | 34.4 | 30.9 | 32.7 |
| IC305179 | G90 | 66.3 | 74.5 | 70.4 | 46.1 | 47.1 | 46.6 | 150 | 142 | 146 | 24.8 | 24.0 | 24.4 | 15.9 | 14.9 | 15.4 | 38.3 | 35.3 | 36.8 |
| IC421767 | G91 | 108 | 93.0 | 101 | 55.2 | 46.0 | 50.6 | 162 | 155 | 159 | 23.3 | 24.8 | 24.1 | 19.0 | 16.0 | 17.5 | 50.5 | 48.7 | 49.6 |
| IC024830 | G92 | 98.3 | 102 | 100 | 52.3 | 46.3 | 49.3 | 161 | 168 | 165 | 21.5 | 24.7 | 23.1 | 17.1 | 17.3 | 17.2 | 49.0 | 45.0 | 47.0 |
| IC331450 | G93 | 71.5 | 84.2 | 77.8 | 33.6 | 31.9 | 32.7 | 151 | 166 | 158 | 18.4 | 20.2 | 19.3 | 17.4 | 18.1 | 17.8 | 39.1 | 36.3 | 37.7 |
| IC625694 | G94 | 50.9 | 61.9 | 56.4 | 36.9 | 34.2 | 35.6 | 146 | 155 | 150 | 22.5 | 24.5 | 23.5 | 19.4 | 19.6 | 19.5 | 34.0 | 30.2 | 32.1 |
| IC351407 | G95 | 72.3 | 84.0 | 78.2 | 48.2 | 35.3 | 41.7 | 144 | 136 | 140 | 23.5 | 24.9 | 24.2 | 8.41 | 6.80 | 7.60 | 32.2 | 28.3 | 30.3 |
| IC406517 | G96 | 61.1 | 71.9 | 66.5 | 46.6 | 35.7 | 41.2 | 146 | 142 | 144 | 23.6 | 24.7 | 24.2 | 8.88 | 9.17 | 9.02 | 34.4 | 30.0 | 32.2 |
| IC467707 | G97 | 70.4 | 63.1 | 66.8 | 40.9 | 32.6 | 36.8 | 133 | 141 | 137 | 23.1 | 20.9 | 22.0 | 12.1 | 10.6 | 11.3 | 60.6 | 52.3 | 56.5 |
| IC550522 | G98 | 65.5 | 51.9 | 58.7 | 44.7 | 34.5 | 39.6 | 140 | 135 | 138 | 22.6 | 21.4 | 22.0 | 9.66 | 6.27 | 7.96 | 28.4 | 25.2 | 26.8 |
| IC550523 | G99 | 62.6 | 52.8 | 57.7 | 37.1 | 33.2 | 35.1 | 136 | 133 | 135 | 23.2 | 23.6 | 23.4 | 6.05 | 5.20 | 5.62 | 18.8 | 23.8 | 21.3 |
|  | **Grand Mean (GM)** | 62.8 | 70.6 | 66.7 | 39.2 | 36.7 | 38.0 | 162 | 159 | 160 | 21.6 | 22.1 | 21.9 | 16.0 | 14.7 | 15.4 | 47.4 | 42.5 | 45.0 |
|  | **Min** | 29.4 | 39.1 | 34.3 | 19.4 | 21.7 | 20.6 | 133 | 133 | 134 | 18.3 | 18.1 | 18.8 | 6.05 | 5.20 | 5.62 | 18.6 | 16.7 | 19.8 |
|  | **Max** | 131 | 130 | 129 | 74.2 | 69.8 | 71.0 | 252 | 243 | 247 | 25.0 | 25.7 | 24.8 | 23.6 | 21.6 | 22.5 | 86.4 | 74.4 | 80.4 |
