# Supplementary material for: Understanding G × E Interaction for Nutritional and Antinutritional Factors in a Diverse Panel of Vigna stipulacea (Lam.) Kuntz Germplasm Tested Over the Locations
Source: Front Plant Sci. 2021 Dec 13;12:766645. doi: 10.3389/fpls.2021.766645 (PMC8710513; doi:10.3389/fpls.2021.766645)
Supplement: Supplementary file 3 [file Table_2.DOCX]

**Table S2 Percent of RDA available for male and female for Fe, Zn, Calcium and Protein among the *Vigna stipulacea***

| **Genotypes** | **Fe** | | | | **Zn** | | | | **Ca** | | **Protein** | | | |
| --- | --- | --- | --- | --- | --- | --- | --- | --- | --- | --- | --- | --- | --- | --- |
|  | **Loc1** | | **Loc2** | | **Loc1** | | **Loc2** | | **Loc1** | **Loc2** | **Loc1** | | **Loc2** | |
|  | **Availability of % RDA from 100 g *Vigna stipulacea*** | | | | | | | | | | | | | |
|  | Male | Female | Male | Female | Male | Female | Male | Female | Male  /Female | Male  /Female | Male | Female | Male | Female |
| G1 | 77.9 | 34.6 | 75.0 | 33.3 | 33.7 | 46.4 | 20.2 | 27.8 | 1.55 | 1.53 | 40.9 | 49.8 | 42.1 | 51.3 |
| G2 | 164 | 72.8 | 158 | 70.4 | 32.2 | 44.3 | 24.3 | 33.4 | 1.61 | 1.56 | 40.1 | 48.8 | 43.4 | 52.9 |
| G3 | 133 | 59.0 | 163 | 72.3 | 32.2 | 44.2 | 23.8 | 32.7 | 1.58 | 1.56 | 36.5 | 44.4 | 42.7 | 51.9 |
| G4 | 108 | 47.8 | 84.2 | 37.4 | 30.2 | 41.5 | 25.8 | 35.5 | 1.48 | 1.52 | 36.3 | 44.2 | 35.9 | 43.7 |
| G5 | 91.9 | 40.8 | 85.3 | 37.9 | 31.2 | 42.9 | 23.9 | 32.9 | 1.62 | 1.60 | 38.8 | 47.2 | 40.0 | 48.6 |
| G6 | 77.9 | 34.6 | 66.0 | 29.4 | 29.8 | 41.0 | 28.2 | 38.8 | 1.36 | 1.42 | 39.5 | 48.0 | 36.2 | 44.1 |
| G7 | 101 | 44.9 | 91.9 | 40.8 | 26.8 | 36.9 | 30.8 | 42.3 | 1.57 | 1.43 | 42.5 | 51.8 | 46.0 | 55.9 |
| G8 | 55.7 | 24.8 | 48.9 | 21.7 | 30.4 | 41.8 | 26.5 | 36.4 | 1.64 | 1.53 | 40.5 | 49.3 | 39.1 | 47.6 |
| G9 | 61.8 | 27.4 | 77.3 | 34.4 | 29.4 | 40.4 | 23.6 | 32.4 | 1.66 | 1.67 | 41.9 | 51.0 | 35.6 | 43.4 |
| G10 | 47.5 | 21.1 | 56.1 | 24.9 | 30.7 | 42.2 | 28.6 | 39.4 | 1.70 | 1.69 | 38.2 | 46.5 | 42.6 | 51.9 |
| G11 | 67.7 | 30.1 | 81.5 | 36.2 | 32.7 | 45.0 | 31.2 | 42.9 | 1.74 | 1.67 | 41.5 | 50.5 | 36.6 | 44.6 |
| G12 | 107 | 47.4 | 125 | 55.6 | 30.2 | 41.5 | 26.9 | 36.9 | 1.69 | 1.69 | 40.1 | 48.9 | 43.0 | 52.4 |
| G13 | 85.6 | 38.0 | 104 | 46.1 | 30.2 | 41.5 | 25.5 | 35.0 | 1.70 | 1.70 | 41.8 | 50.9 | 45.0 | 54.8 |
| G14 | 48.6 | 21.6 | 56.0 | 24.9 | 20.1 | 27.6 | 22.2 | 30.5 | 1.36 | 1.47 | 40.2 | 48.9 | 35.8 | 43.6 |
| G15 | 78.1 | 34.7 | 95.5 | 42.4 | 33.8 | 46.5 | 29.3 | 40.3 | 1.77 | 1.58 | 43.6 | 53.1 | 42.0 | 51.2 |
| G16 | 85.2 | 37.9 | 105 | 46.7 | 37.9 | 52.1 | 33.3 | 45.8 | 1.79 | 1.73 | 43.6 | 53.1 | 41.6 | 50.6 |
| G17 | 74.7 | 33.2 | 85.0 | 37.8 | 32.2 | 44.3 | 41.7 | 57.3 | 1.56 | 1.62 | 39.6 | 48.2 | 38.5 | 46.9 |
| G18 | 107 | 47.3 | 129 | 57.4 | 27.9 | 38.4 | 24.3 | 33.3 | 1.59 | 1.58 | 41.9 | 51.0 | 44.2 | 53.9 |
| G19 | 74.6 | 33.1 | 91.7 | 40.7 | 29.8 | 41.0 | 28.8 | 39.6 | 1.62 | 1.62 | 42.0 | 51.1 | 37.9 | 46.2 |
| G20 | 65.2 | 29.0 | 88.3 | 39.2 | 32.3 | 44.5 | 33.3 | 45.8 | 1.51 | 1.48 | 41.6 | 50.7 | 39.5 | 48.1 |
| G21 | 51.9 | 23.1 | 67.9 | 30.2 | 25.8 | 35.5 | 25.4 | 35.0 | 1.55 | 1.46 | 41.2 | 50.1 | 43.8 | 53.4 |
| G22 | 75.9 | 33.7 | 80.3 | 35.7 | 35.9 | 49.4 | 30.9 | 42.5 | 1.83 | 1.71 | 41.8 | 50.9 | 36.5 | 44.4 |
| G23 | 106 | 47.0 | 128 | 56.8 | 35.1 | 48.2 | 29.7 | 40.9 | 1.82 | 1.81 | 40.9 | 49.8 | 38.5 | 46.8 |
| G24 | 55.8 | 24.8 | 83.5 | 37.1 | 30.5 | 41.9 | 29.7 | 40.9 | 1.66 | 1.61 | 40.1 | 48.8 | 42.5 | 51.8 |
| G25 | 59.0 | 26.2 | 72.2 | 32.1 | 35.3 | 48.5 | 31.6 | 43.4 | 1.72 | 1.71 | 39.4 | 47.9 | 35.6 | 43.4 |
| G26 | 88.5 | 39.3 | 107 | 47.6 | 31.1 | 42.7 | 30.0 | 41.3 | 1.74 | 1.61 | 39.8 | 48.4 | 42.2 | 51.4 |
| G27 | 71.5 | 31.8 | 94.1 | 41.8 | 31.2 | 42.9 | 28.9 | 39.7 | 1.75 | 1.71 | 43.4 | 52.8 | 44.5 | 54.1 |
| G28 | 57.9 | 25.7 | 80.8 | 35.9 | 29.3 | 40.3 | 27.5 | 37.8 | 1.72 | 1.67 | 34.7 | 42.2 | 37.1 | 45.1 |
| G29 | 47.3 | 21.0 | 55.8 | 24.8 | 26.0 | 35.7 | 28.6 | 39.3 | 1.81 | 1.73 | 42.0 | 51.1 | 42.3 | 51.5 |
| G30 | 53.7 | 23.9 | 70.9 | 31.5 | 28.1 | 38.6 | 31.9 | 43.9 | 1.74 | 1.69 | 41.5 | 50.5 | 38.4 | 46.8 |
| G31 | 47.2 | 21.0 | 56.7 | 25.2 | 17.7 | 24.3 | 19.8 | 27.2 | 1.35 | 1.33 | 41.0 | 49.9 | 36.8 | 44.8 |
| G32 | 39.1 | 17.4 | 53.5 | 23.8 | 19.1 | 26.2 | 22.9 | 31.4 | 1.74 | 1.71 | 40.4 | 49.2 | 36.6 | 44.6 |
| G33 | 93.9 | 41.7 | 127 | 56.6 | 29.5 | 40.6 | 20.5 | 28.2 | 1.71 | 1.70 | 36.6 | 44.5 | 39.0 | 47.5 |
| G34 | 40.6 | 18.0 | 63.4 | 28.2 | 25.8 | 35.4 | 29.0 | 39.9 | 1.71 | 1.72 | 33.8 | 41.1 | 36.1 | 43.9 |
| G35 | 57.2 | 25.4 | 81.2 | 36.1 | 22.0 | 30.2 | 26.1 | 35.9 | 1.62 | 1.59 | 38.0 | 46.3 | 40.5 | 49.3 |
| G36 | 76.2 | 33.9 | 91.3 | 40.6 | 23.9 | 32.8 | 26.9 | 37.0 | 1.65 | 1.64 | 38.5 | 46.8 | 41.5 | 50.5 |
| G37 | 137 | 60.9 | 153 | 67.9 | 32.0 | 43.9 | 28.5 | 39.2 | 1.74 | 1.72 | 41.5 | 50.5 | 38.0 | 46.2 |
| G38 | 80.4 | 35.7 | 102 | 45.2 | 31.7 | 43.6 | 29.6 | 40.8 | 1.72 | 1.66 | 40.0 | 48.7 | 44.2 | 53.9 |
| G39 | 52.4 | 23.3 | 69.3 | 30.8 | 31.0 | 42.6 | 28.8 | 39.5 | 1.75 | 1.74 | 35.0 | 42.6 | 36.1 | 43.9 |
| G40 | 76.2 | 33.8 | 82.0 | 36.4 | 28.0 | 38.5 | 29.7 | 40.9 | 1.78 | 1.84 | 36.8 | 44.8 | 39.9 | 48.6 |
| G41 | 66.8 | 29.7 | 91.3 | 40.6 | 25.9 | 35.6 | 28.4 | 39.1 | 1.73 | 1.72 | 33.3 | 40.5 | 35.3 | 43.0 |
| G42 | 49.7 | 22.1 | 56.3 | 25.0 | 28.1 | 38.6 | 27.5 | 37.8 | 1.51 | 1.52 | 35.2 | 42.9 | 37.0 | 45.0 |
| G43 | 61.9 | 27.5 | 75.6 | 33.6 | 26.3 | 36.2 | 25.3 | 34.8 | 1.55 | 1.53 | 35.2 | 42.8 | 33.7 | 41.0 |
| G44 | 53.1 | 23.6 | 54.8 | 24.4 | 31.1 | 42.8 | 28.4 | 39.0 | 1.43 | 1.41 | 43.5 | 53.0 | 41.4 | 50.4 |
| G45 | 89.3 | 39.7 | 129 | 57.5 | 43.1 | 59.3 | 33.1 | 45.5 | 1.55 | 1.53 | 34.9 | 42.4 | 35.0 | 42.6 |
| G46 | 50.5 | 22.5 | 68.7 | 30.5 | 30.6 | 42.0 | 28.8 | 39.6 | 1.55 | 1.49 | 36.2 | 44.1 | 34.4 | 41.9 |
| G47 | 52.9 | 23.5 | 69.3 | 30.8 | 29.2 | 40.2 | 28.1 | 38.6 | 1.54 | 1.49 | 36.6 | 44.5 | 38.0 | 46.3 |
| G48 | 60.3 | 26.8 | 79.1 | 35.2 | 33.8 | 46.4 | 32.6 | 44.9 | 1.54 | 1.53 | 37.1 | 45.1 | 38.3 | 46.7 |
| G49 | 85.8 | 38.1 | 100 | 44.5 | 47.4 | 65.2 | 39.6 | 54.5 | 1.57 | 1.48 | 41.6 | 50.7 | 43.3 | 52.8 |
| G50 | 55.9 | 24.9 | 71.1 | 31.6 | 31.1 | 42.8 | 29.9 | 41.0 | 1.72 | 1.66 | 39.0 | 47.5 | 41.7 | 50.8 |
| G51 | 52.9 | 23.5 | 55.6 | 24.7 | 35.4 | 48.7 | 31.8 | 43.7 | 1.62 | 1.61 | 35.0 | 42.6 | 36.7 | 44.7 |
| G52 | 63.6 | 28.3 | 67.8 | 30.2 | 33.1 | 45.5 | 28.9 | 39.8 | 1.61 | 1.58 | 35.6 | 43.4 | 39.8 | 48.5 |
| G53 | 85.5 | 38.0 | 94.9 | 42.2 | 23.4 | 32.2 | 30.0 | 41.3 | 1.64 | 1.64 | 34.7 | 42.2 | 38.6 | 47.0 |
| G54 | 66.4 | 29.5 | 95.0 | 42.2 | 30.9 | 42.5 | 28.6 | 39.3 | 1.64 | 1.57 | 34.8 | 42.3 | 33.3 | 40.5 |
| G55 | 52.9 | 23.5 | 66.8 | 29.7 | 29.3 | 40.3 | 28.9 | 39.8 | 1.52 | 1.49 | 36.0 | 43.8 | 39.9 | 48.6 |
| G56 | 36.7 | 16.3 | 49.2 | 21.9 | 21.9 | 30.2 | 30.8 | 42.3 | 1.53 | 1.47 | 37.9 | 46.1 | 34.9 | 42.5 |
| G57 | 55.5 | 24.7 | 70.8 | 31.5 | 30.8 | 42.4 | 30.2 | 41.5 | 1.54 | 1.52 | 43.3 | 52.7 | 38.6 | 47.0 |
| G58 | 44.4 | 19.7 | 58.5 | 26.0 | 23.8 | 32.8 | 30.2 | 41.5 | 1.53 | 1.48 | 37.0 | 45.1 | 41.2 | 50.1 |
| G59 | 54.5 | 24.2 | 67.1 | 29.8 | 34.7 | 47.8 | 31.3 | 43.0 | 2.21 | 2.01 | 38.2 | 46.5 | 43.1 | 52.5 |
| G60 | 96.2 | 42.8 | 101 | 44.9 | 37.2 | 51.1 | 34.6 | 47.5 | 2.52 | 2.43 | 36.3 | 44.2 | 38.3 | 46.6 |
| G61 | 75.9 | 33.7 | 77.5 | 34.4 | 37.5 | 51.6 | 30.6 | 42.1 | 1.95 | 1.87 | 34.9 | 42.4 | 40.2 | 49.0 |
| G62 | 77.5 | 34.4 | 77.6 | 34.5 | 37.2 | 51.2 | 35.6 | 49.0 | 1.56 | 1.52 | 39.7 | 48.4 | 34.7 | 42.2 |
| G63 | 161 | 71.7 | 134 | 59.7 | 50.4 | 69.3 | 39.3 | 54.0 | 1.54 | 1.52 | 43.2 | 52.5 | 38.3 | 46.6 |
| G64 | 76.6 | 34.0 | 102 | 45.1 | 52.2 | 71.7 | 38.7 | 53.2 | 1.56 | 1.54 | 44.6 | 54.3 | 43.6 | 53.1 |
| G65 | 64.3 | 28.6 | 83.7 | 37.2 | 61.4 | 84.4 | 52.9 | 72.8 | 1.65 | 1.62 | 38.3 | 46.6 | 35.1 | 42.7 |
| G66 | 76.2 | 33.9 | 92.4 | 41.1 | 46.4 | 63.7 | 45.7 | 62.9 | 1.65 | 1.66 | 34.9 | 42.5 | 38.2 | 46.5 |
| G67 | 61.5 | 27.4 | 69.0 | 30.7 | 64.9 | 89.3 | 63.5 | 87.3 | 1.51 | 1.43 | 38.2 | 46.5 | 41.4 | 50.4 |
| G68 | 95.6 | 42.5 | 104 | 46.2 | 58.5 | 80.4 | 58.3 | 80.2 | 1.52 | 1.46 | 36.3 | 44.2 | 38.5 | 46.9 |
| G69 | 92.5 | 41.1 | 97.0 | 43.1 | 43.2 | 59.4 | 47.0 | 64.7 | 1.61 | 1.60 | 36.8 | 44.7 | 40.0 | 48.7 |
| G70 | 104 | 46.4 | 117 | 51.8 | 37.1 | 51.0 | 42.0 | 57.8 | 1.46 | 1.43 | 36.9 | 44.9 | 38.8 | 47.2 |
| G71 | 66.6 | 29.6 | 76.8 | 34.2 | 38.5 | 53.0 | 37.4 | 51.5 | 1.61 | 1.60 | 34.8 | 42.4 | 32.3 | 39.4 |
| G72 | 89.1 | 39.6 | 109 | 48.5 | 41.6 | 57.2 | 38.1 | 52.4 | 1.55 | 1.56 | 40.6 | 49.4 | 44.0 | 53.5 |
| G73 | 76.7 | 34.1 | 89.9 | 39.9 | 41.4 | 57.0 | 38.6 | 53.0 | 1.46 | 1.42 | 35.0 | 42.6 | 41.4 | 50.4 |
| G74 | 158 | 70.1 | 151 | 67.1 | 49.3 | 67.8 | 43.6 | 60.0 | 1.51 | 1.52 | 40.1 | 48.9 | 43.9 | 53.5 |
| G75 | 118 | 52.5 | 128 | 57.0 | 58.6 | 80.6 | 49.7 | 68.3 | 1.53 | 1.52 | 34.4 | 41.8 | 38.1 | 46.3 |
| G76 | 134 | 59.6 | 117 | 51.9 | 67.5 | 92.8 | 61.6 | 84.8 | 1.53 | 1.49 | 33.4 | 40.6 | 35.4 | 43.1 |
| G77 | 133 | 59.2 | 117 | 51.9 | 48.6 | 66.8 | 43.7 | 60.0 | 1.43 | 1.48 | 37.1 | 45.2 | 36.9 | 44.9 |
| G78 | 86.4 | 38.4 | 89.9 | 39.9 | 35.8 | 49.2 | 38.6 | 53.0 | 1.70 | 1.73 | 34.6 | 42.2 | 38.2 | 46.5 |
| G79 | 84.4 | 37.5 | 91.5 | 40.7 | 33.0 | 45.4 | 44.8 | 61.6 | 1.61 | 1.55 | 42.0 | 51.2 | 43.4 | 52.9 |
| G80 | 94.8 | 42.1 | 102 | 45.5 | 41.2 | 56.7 | 46.2 | 63.5 | 1.72 | 1.71 | 37.8 | 46.1 | 41.8 | 50.9 |
| G81 | 64.4 | 28.6 | 77.4 | 34.4 | 51.3 | 70.5 | 46.6 | 64.0 | 1.64 | 1.61 | 37.3 | 45.4 | 41.7 | 50.8 |
| G82 | 65.2 | 29.0 | 80.6 | 35.8 | 37.4 | 51.4 | 47.9 | 65.8 | 1.66 | 1.63 | 36.0 | 43.8 | 37.6 | 45.8 |
| G83 | 60.3 | 26.8 | 64.9 | 28.9 | 46.1 | 63.4 | 41.5 | 57.0 | 1.65 | 1.63 | 36.8 | 44.8 | 42.3 | 51.5 |
| G84 | 71.5 | 31.8 | 77.4 | 34.4 | 28.7 | 39.4 | 29.8 | 41.0 | 1.49 | 1.45 | 37.8 | 46.0 | 42.5 | 51.7 |
| G85 | 76.1 | 33.8 | 89.3 | 39.7 | 34.2 | 47.0 | 30.1 | 41.3 | 1.52 | 1.53 | 36.1 | 43.9 | 37.9 | 46.1 |
| G86 | 61.8 | 27.5 | 66.6 | 29.6 | 38.6 | 53.1 | 31.0 | 42.6 | 1.53 | 1.46 | 33.1 | 40.3 | 35.1 | 42.7 |
| G87 | 92.8 | 41.3 | 79.9 | 35.5 | 48.5 | 66.6 | 44.7 | 61.5 | 1.65 | 1.71 | 32.8 | 39.9 | 36.1 | 44.0 |
| G88 | 61.7 | 27.4 | 64.9 | 28.9 | 39.3 | 54.0 | 37.1 | 51.0 | 1.72 | 1.76 | 32.7 | 39.8 | 34.7 | 42.2 |
| G89 | 64.1 | 28.5 | 79.5 | 35.4 | 52.9 | 72.7 | 41.9 | 57.7 | 1.46 | 1.53 | 38.1 | 46.4 | 42.0 | 51.2 |
| G90 | 82.9 | 36.8 | 93.1 | 41.4 | 41.9 | 57.6 | 42.9 | 58.9 | 1.50 | 1.42 | 44.2 | 53.8 | 42.9 | 52.3 |
| G91 | 135 | 60.1 | 116 | 51.7 | 50.2 | 69.0 | 41.9 | 57.5 | 1.62 | 1.55 | 41.7 | 50.7 | 44.2 | 53.9 |
| G92 | 123 | 54.6 | 128 | 56.8 | 47.5 | 65.3 | 42.1 | 57.9 | 1.61 | 1.68 | 38.4 | 46.7 | 44.2 | 53.8 |
| G93 | 89.3 | 39.7 | 105 | 46.8 | 30.5 | 42.0 | 29.0 | 39.9 | 1.51 | 1.66 | 32.8 | 39.9 | 36.1 | 44.0 |
| G94 | 63.6 | 28.3 | 77.4 | 34.4 | 33.6 | 46.2 | 31.1 | 42.7 | 1.46 | 1.55 | 40.2 | 48.9 | 43.8 | 53.3 |
| G95 | 90.4 | 40.2 | 105 | 46.7 | 43.8 | 60.3 | 32.1 | 44.1 | 1.44 | 1.36 | 42.0 | 51.1 | 44.5 | 54.2 |
| G96 | 76.3 | 33.9 | 89.9 | 40.0 | 42.4 | 58.3 | 32.5 | 44.7 | 1.46 | 1.42 | 42.1 | 51.2 | 44.2 | 53.8 |
| G97 | 88.0 | 39.1 | 78.8 | 35.0 | 37.2 | 51.1 | 29.6 | 40.8 | 1.33 | 1.41 | 41.2 | 50.1 | 37.3 | 45.4 |
| G98 | 81.8 | 36.4 | 64.9 | 28.9 | 40.6 | 55.9 | 31.3 | 43.1 | 1.40 | 1.35 | 40.3 | 49.1 | 38.1 | 46.4 |
| G99 | 78.2 | 34.8 | 66.0 | 29.3 | 33.7 | 46.4 | 30.2 | 41.5 | 1.36 | 1.33 | 41.4 | 50.4 | 42.1 | 51.3 |
| **Average** | **78.4** | **34.9** | **88.3** | **39.2** | **35.7** | **49.1** | **33.4** | **45.9** | **1.62** | **1.59** | **38.6** | **46.9** | **39.5** | **48.1** |
| **Min** | **36.7** | **16.3** | **48.9** | **21.7** | **17.7** | **24.3** | **19.8** | **27.2** | **1.33** | **1.33** | **32.7** | **39.8** | **32.3** | **39.4** |
| **Max** | **164** | **72.8** | **163** | **72.3** | **67.5** | **92.8** | **63.5** | **87.3** | **2.52** | **2.43** | **44.6** | **54.3** | **46.0** | **55.9** |
